# Microbiome and ecotypic adaption of *Holcus lanatus* (L.) to extremes of its soil pH range, investigated through transcriptome sequencing

**DOI:** 10.1186/s40168-018-0434-3

**Published:** 2018-03-20

**Authors:** Ellen Young, Manus Carey, Andrew A. Meharg, Caroline Meharg

**Affiliations:** Institute for Global Food Security, Queens University Belfast, David Keir Building, Belfast, BT9 5BN Northern Ireland, UK

**Keywords:** *Holcus lanatus*, Eukaryotic microbiome, Meta-transcriptomics, Community composition, Gene expression, Gene function, Edaphic stress

## Abstract

**Background:**

Plants can adapt to edaphic stress, such as nutrient deficiency, toxicity and biotic challenges, by controlled transcriptomic responses, including microbiome interactions. Traditionally studied in model plant species with controlled microbiota inoculation treatments, molecular plant-microbiome interactions can be functionally investigated via RNA-Seq. Complex, natural plant-microbiome studies are limited, typically focusing on microbial rRNA and omitting functional microbiome investigations, presenting a fundamental knowledge gap. Here, root and shoot meta-transcriptome analyses, in tandem with shoot elemental content and root staining, were employed to investigate transcriptome responses in the wild grass *Holcus lanatus* and its associated natural multi-species eukaryotic microbiome. A full factorial reciprocal soil transplant experiment was employed, using plant ecotypes from two widely contrasting natural habitats, acid bog and limestone quarry soil, to investigate naturally occurring, and ecologically meaningful, edaphically driven molecular plant-microbiome interactions.

**Results:**

Arbuscular mycorrhizal (AM) and non-AM fungal colonization was detected in roots in both soils. Staining showed greater levels of non-AM fungi, and transcriptomics indicated a predominance of Ascomycota-annotated genes. Roots in acid bog soil were dominated by *Phialocephala*-annotated transcripts, a putative growth-promoting endophyte, potentially involved in N nutrition and ion homeostasis. Limestone roots in acid bog soil had greater expression of other Ascomycete genera and Oomycetes and lower expression of *Phialocephala*-annotated transcripts compared to acid ecotype roots, which corresponded with reduced induction of pathogen defense processes, particularly lignin biosynthesis in limestone ecotypes. Ascomycota dominated in shoots and limestone soil roots, but *Phialocephala*-annotated transcripts were insignificant, and no single Ascomycete genus dominated. *Fusarium*-annotated transcripts were the most common genus in shoots, with *Colletotrichum* and *Rhizophagus* (AM fungi) most numerous in limestone soil roots. The latter coincided with upregulation of plant genes involved in AM symbiosis initiation and AM-based P acquisition in an environment where P availability is low.

**Conclusions:**

Meta-transcriptome analyses provided novel insights into *H*. *lanatus* transcriptome responses, associated eukaryotic microbiota functions and taxonomic community composition. Significant edaphic and plant ecotype effects were identified, demonstrating that meta-transcriptome-based functional analysis is a powerful tool for the study of natural plant-microbiome interactions.

**Electronic supplementary material:**

The online version of this article (10.1186/s40168-018-0434-3) contains supplementary material, which is available to authorized users.

## Background

Extremes of soil pH present strong selection pressures, particularly relating to nutrient availabilities. Soils with pH < 5.5 cause Al, Fe, Mn and H toxicities and simultaneous P, N and base cation deficiencies, resulting in inhibition of root growth and poor productivity [1]. Neutral to alkaline soils are limited in Fe, Mn and P availability [1]. Decreasing soil bacterial activity with increasing soil acidity regulates N availability, with nitrate dominating at neutral to high pHs, ammonium at low pHs, and amino acids at extreme low pH [2]. Soil pH also influences edaphic bacterial and fungal community compositions, including root-colonizing arbuscular mycorrhiza (AM) and non-AM fungi, with disparate edaphic conditions driving differences in root colonization within the same host species [3–5]. Non-AM fungi are abundant in all soils, including acid peatland [4], while AM fungi have been shown to occur at lower frequency in lower pH and waterlogged soils [5].

Components of the soil microbiome interact with plants in beneficial, neutral or pathogenic manners. AM and root endophytes have been shown to increase nutrient uptake, particularly P [6–8], and plants can actively encourage AM fungal colonization under low nutrient stress conditions [9]. Equally, fungi can influence gene expression in plants. Fungal induction of plant lipoxygenase and its associated pathway has been linked to fungal-mediated tolerance traits, and plant defenses primed or boosted by fungi include antioxidant, phenol and flavonoid production and toxic metal chelation [10–13]. Fungal colonization can improve plant resistance to pathogen infection [13], salt stress [12] and toxic metal(loid) stress [11, 13, 14], the latter of which is common on acidic soils due to higher bioavailability of Al, Fe and Mn [2, 15]. Pathogenic fungal elicitors cause initiation of plant defensive responses upon detection [16, 17], but necrotrophic fungi can in turn manipulate plant defenses to facilitate initial infection, with further manipulation of the oxidative burst response to continue colonization [18].

Given these interactions, characterizing host-microbiome relationships therefore require analysis of gene expression and functional responses from both components, plant and fungal. Furthermore, given that plant-microbiome interactions are strongly influenced by edaphic factors, they can be considered key to the understanding of plant edaphic stress response and crucial for our understanding of plant adaptation to environmental change [19]. Assessment of soil and root microbiomes has traditionally been taxonomy based, using amplicon sequencing of the rRNA operon [20, 21], which, as normally DNA based, cannot distinguish between metabolically active and dormant components of the plant-microbiome system or provide information about functional roles. Furthermore, as plant-microbiome interactions involve multiple microbial species, there must be a high level of functional redundancy, with a range of species fulfilling the same or similar functions in different environmental niches, which a gene expression-based investigation can address. For prokaryotes, PICRUSt (phylogenetic investigation of communities by reconstruction of unobserved states) analysis [22] allows inference of metagenomes and metabolic potential from amplicon sequencing data, but similar analyses are not available for fungi and other eukaryotic microbes. Even for prokaryotes, gene expression data is the only way to measure true activity. As next-generation sequencing (NGS), via sequencing of polyA-selected RNAs, provides a technology that can capture gene expression of all eukaryotes in any one sample, this is a convenient way to investigate host and eukaryotic microbiomes in tandem. Published annotated genomes and protein databases for plants, fungi and protists facilitate a functional meta-transcriptomic approach that can uncover eukaryotic microbiome function in the context of plant transcriptome analysis. Such integrated analyses can further the holistic understanding of edaphic stress, plant ecotype adaptation and ecosystem function [23, 24].

Traditionally, microbial genome alignment is used to remove contaminating non-plant transcripts for a plant-centered analysis, but gene expression and function of the host and microbes are increasingly being investigated in tandem [25]. However, this typically involves the study of model plants in controlled interactions with specific fungi, to facilitate alignment of reads to published genomes for assignment of plant versus microbiome transcripts [26–32]. Studies of more complex, ecologically relevant and genetically diverse non-model plant-microbiome functional interactions are lacking. This current investigation addresses this gap in our knowledge. Using a meta-transcriptomics approach, we have analysed distinct genotypes of *Holcus lanatus* (L.) selected from two widely contrasting edaphic environments.

The wild grass *H*. *lanatus* colonizes a wide range of soils with strongly contrasting abiotic stresses including acid bogs, calcareous soils, saline soils and metal(loid)-contaminated mine spoils [33]. Such an adaptive range implies selection for different ecotypes, involving genetic changes under differential selection pressures [34, 35], and genetic changes associated with edaphic stress adaptation and plasticity have been observed in this species [36–38]. *H*. *lanatus* forms fungal associations [33], some of which facilitate survival and adaptation to edaphic stress [39]. Just as *H*. *lanatus* exhibits ecotypic variation between different environments [37], so too does its microbiome, including root fungal composition [40]. This most likely facilitates the development of a range of beneficial environment-specific plant-microbiome interactions. Because of its inherent plasticity, *H*. *lanatus* is an ideal species to study plant-microbiome adaptations to edaphic stress, including simultaneous investigation of plant and eukaryotic microbiome responses to variations in soil characteristics influenced by pH, as presented in this study. Extremes of pH present a wide range of challenges to plants [41], and *H*. *lanatus* is tolerant of a wide soil pH range, from at least 3.5 to 8 [33, 34]. This is exemplified by the populations used in this current study, collected from an acid bog of pH 3.5 (mainly composed of organic material) and a limestone quarry soil of pH 7.5 (calcareous clay, with low level of organic matter content). Biotic factors, particularly soil fungal communities, will contrast in these habitats and will involve beneficial, neutral and detrimental soil-specific plant-microbial interactions [1].

In this study, 10 *H*. *lanatus* genotypes, 5 acid bog and 5 limestone quarry, were investigated using a full factorial reciprocal soils of origin transplant experiment. The aim was to capture the natural genetic diversity in the host and microbiome via RNA-Seq analysis of root and shoot of this species. Root staining was employed to validate AM and non-AM fungal colonization levels, and shoot elemental content to aid interpretation in the context of nutrient ion homeostasis and edaphic stress response. To our knowledge, no other study to date has investigated the overall functional and taxonomic diversity of ecologically relevant plant root and shoot eukaryotic microbiomes within the ecological context of ecotype plasticity and edaphic stress adaptation.

## Methods

### Plants and soils

Intact *H*. *lanatus* plants (shoot and root ball) were collected along with topsoil from two locations in Northern Ireland; a disused limestone quarry, pH 7.5, Map. Ref. NR 23472 02816; and an acidic peat bog, pH 3.5, Map. Ref. NW 02918 19660. Plants were collected at least 2 m apart, ensuring each plant represents a unique genotype of that particular habitat. *H*. *lanatus* can be propagated from unrooted tillers as roots develop from tiller basal nodes. Unrooted tillers were planted into compost (John Innes no.2) and maintained in a growth chamber (Memmert, Germany) at 20 °C, 10000 LUX light intensity and 12 h day:night cycle, irrigated to water holding capacity. Therefore, all tillers used in subsequent experimentation originated from under the same conditions.

For the full factorial reciprocal transplant experiment, individual unrooted tillers from 5 acid bog and 5 limestone quarry ecotypes were transplanted onto acid bog and limestone soils, in a fully reciprocal transplantation design. Replication was at genotype level, allowing representation of natural population variation. These soil-grown plants were kept under the same growth chamber conditions as for tiller generation. Treatment coding is as follows: lowercase “*a*” is for plant ecotype collected from acid bog soil, and “*l*” from limestone soil. Uppercase “*A*” indicates acid bog peat as the growth medium, and “*L*” for limestone soil medium. Plants were harvested after 7 weeks, roots and shoots separated, rinsed in deionized water, frozen in liquid nitrogen and stored at − 80 °C.

### Physiochemical analysis of the soils and plants

Soils were oven dried (70 °C) and milled. Milled soil was compacted into 32-mm cylindrical disks of ≥ 6 mm width and processed using a Rigaku NEXCG energy dispersive X-ray fluorescence spectrometer (Rigaku, Japan), in the presence of helium, to ascertain elemental content. Inductively coupled plasma-mass spectroscopy (ICP-MS analysis), using an iCAP Qc ICP-MS (ThermoFisher Scientific, USA), was used to compliment XRF analysis, as XRF better quantifies macro-elements and ICP-MS micro-elements. For ICP-MS, dried and milled soils were treated with two acid mixes: 5 ml of 69% nitric acid or 3 ml 69% nitric acid plus 2 ml 37% hydrochloric acid, both acids of Aristar grade. Samples were digested in a Mars6 240/250 microwave (CEM Corporation, USA) at 200 °C for 30 min after a one-stage 15 min heating ramp to 165 °C. Element recovery was compared to a soil certified reference material (CRM), NCS ZC73007 and ISE921, (LGC Standards), and the best quantification method for each element, according to CRM recovery, was reported. ICP-MS was also conducted on soil-grown shoot material. Shoots were freeze-dried, milled and digested in a nitric acid and peroxide solution as detailed in Signes-Pastor et al. [42]. Organic matter content was measured via loss of ignition (LoI), quantifying weight loss on controlled burning of soils placed in a porcelain crucible and treated to 24 h to a temperature of 400 °C overnight in a muffle furnace. pH was determined from soil slurries produced from milled soil and distilled water using a pH probe. Statistical analyses using GLMs were conducted in Minitab13 (Minitab, USA). Where normality tests on residuals indicated non-normality, data was log_2_ transformed.

### Microscopy-based assessment of root fungal colonization

The reciprocal soil transplantation experiment was repeated using six plants per treatment to assess root colonization rates of AM and non-AM fungi. Additionally, four acid bog and four limestone quarry plants were collected and maintained on their soils of origin to assess natural fungal colonization levels. Roots were cleared in 10% potassium hydroxide (Sigma-Aldrich), rinsed with 10% acetic acid (Sigma-Aldrich), stained with a 10% ink solution (Sheaffer Skrip Black, Sheaffer, USA) and mounted in lactoglycerol. The presence and percent colonization of AM and non-AM fungi was assessed based on 100 intersections per plant using the magnified intersection method [43] with an Olympus (Tokyo, Japan) BX43F microscope. Colonization levels were statistically analysed in Minitab using ranked data due to non-normality, employing GLMs and two-sample *t* tests and plotted using SigmaPlot (SigmaPlot, USA).

### RNA extraction and sequencing

Soil-grown shoots were homogenized to a fine powder under liquid nitrogen and Lysing Matrix D (MP Biomedicals, USA) using two 20-s runs on a Precellys 24-Dual beadbeater (Bertin Technologies, France). Soil-grown roots were pre-ground using 1.5-ml microcentrifuge pestles (Sigma-Aldrich, USA) and ≤ 106-μm acid washed glass beads (Sigma-Aldrich) before homogenization using Lysing Matrix A (MP Biomedicals) with the same bead-beater conditions as shoots. RNA was extracted using the RNeasy Plant Mini Kit (QIAGEN, Germany) incorporating on-column DNase treatment (RNase-free DNase Set, QIAGEN) following the standard protocol with the following amendments: 450 μl Buffer RLT (containing 4.5 μl β-Mercaptoethanol) was added to the powdered plant material and processed for 5 s at 5500 rpm in the bead-beater. The lysate was transferred to a QIAshredder spin column and centrifuged for 2 min at 14000 rpm. RNA was double-eluted using the same eluate and stored at − 80 °C.

RNA quality was ascertained using a Nanodrop 8000 spectrophotometer (ThermoFisher Scientific) and an Agilent 2200 Tape Station (Agilent Technologies, USA). Four samples failed quality checks and were not sequenced. Barcoded 125 bp paired-end libraries (Illumina TruSeq, polyA selected to enrich for eukaryotic mRNA and remove rRNA) were generated and sequenced at the Earlham Institute (UK) on an Illumina HiSeq 2500. Samples were sequenced across four lanes (36 samples from the soil transplant experiment plus 3 additional samples; see Additional file 1). The RNA-Seq data (fastq files) is publicly available in ArrayExpress under accession E-MTAB-4014 at https://www.ebi.ac.uk/arrayexpress/E-MTAB-4014.

### Quality control of sequencing reads

Fastq files were quality checked using Fastqc [44] and Illumina adapters removed with seqtk [45]. Reads were trimmed to remove the first 14 bases, those with Phred quality < 20 from the end of reads, all reads containing any N bases and those with < 100 bases post-trimming using Fastq-mcf [46]. Where a read was discarded, its pair was also discarded.

### Meta-transcriptome assembly, annotation and alignment

Trinity v2.0.6 [47] was used to produce multiple transcriptome assemblies using trimmed paired reads and default settings. Additional plants were added to the transcriptome assembly to increase meta-transcriptome coverage (see Additional file 1). Assembled sequences were sequentially annotated via basic local alignment search tool (BLASTx) [48] using a range of databases and an *e* value cutoff of e−08. The following databases were downloaded from NCBI reference sequences (RefSeq) [49]: plant-refseq release 71 [50], protozoa-refseq release 71 [51] and fungal-refseq release 72 [52]. *Brachypodium distachyon* and *Arabidopsis thaliana* databases were downloaded from AgriGO [53]. The following protein databases, including KOG (EuKaryotic Orthologous Group) annotation files for functional annotation, were downloaded from JGI [54, 55]: *Arabidopsis lyrata* [56], *Rhizophagus irregularis* [57], *Marssonina brunnea* [58], *Colletotrichum graminicola* [59], *Agaricus bisporus* [60] and *Phytophthora soyae* [61]. All assembled transcripts were initially BLASTed against plant-refseq, protozoa-refseq, fungi-refseq and the *Rhizophagus irregularis* protein database, and annotated transcripts were merged with a previously published *H*. *lanatus* 454 transcriptome assembly [38]. Duplicated annotations were removed based on retention of the transcript with the best BLAST score for each primary accession ID. Transcripts were assigned as plant or non-plant based on best BLAST score. Plant-assigned transcripts were further filtered to remove those with plant-refseq gene identity and sequence coverage ≤ 70%. Microbial-assigned transcripts were further filtered to remove those with best microbial annotation gene identity ≤ 70% and sequence coverage ≤ 90%. This resulted in a final annotated reference transcriptome containing plant and microbially assigned transcripts. The best microbial annotation was used to obtain kingdom, phylum and species level information for each microbially assigned transcript. BLASTx against various genome protein databases from the JGI (see above), using an *e* value cutoff of e−08, was subsequently performed, and the most relevant KOG [62] functional annotations for each plant and microbial transcript recorded. Retained plant-assigned transcripts were BLASTed against AgriGO *B*. *distachyon* and *A*. *thaliana* protein databases to provide identifiers for Gene Ontology (GO)-based enrichment analysis.

Paired reads from the 36 soil-grown samples were aligned to the annotated reference transcriptome using Bowtie2 [63], allowing one mismatch in the seed and reporting on all valid alignments. The number of aligned reads per sample was counted using a Perl script. In order to remove transcripts with 0 or very low counts across most samples, the count table was filtered across all 36 samples using edgeR [64] to retain only those transcripts with ≥ 5 counts in at least 3 out of the 36 samples. Remaining expressed microbial transcripts were further BLASTed against the NCBI non-redundant (nr) protein database [65], using an *e* value cutoff of e−08, for a further iteration of taxonomic annotation of microbial transcripts based on best BLAST score. Thus, the final taxonomic annotation for all expressed transcripts was taken from the best hit from a combined BLASTx result (NCBI plant-refseq, NCBI protozoa-refseq, NCBI fungi-refseq, JGI *Rhizophagus irregularis* and NCBI nr).

### Statistical and functional analyses

Differential expression analysis of pair-wise comparisons using the 36 soil-grown samples was conducted using DESeq2 to detect differential expression based on soil type and plant ecotype effects [66]. Separate analysis pipelines were used for identification of differentially expressed plant and microbial transcripts. For differential expression of plant transcripts, one root sample was removed as it showed much lower than average counts for plant-assigned transcripts, and DESeq2 analysis was repeated with 35 samples. Following DESeq2 analysis of plant transcripts, a count of 5 was added to DESeq2 baseMeans for each pair-wise comparison and log_2_fold changes (log_2_FCs) were recalculated to aid removal of significant FC calls from expressed transcripts with low counts. Transcripts were considered significantly differentially expressed if false discovery rate (FDR) < 0.05 and recalculated absolute log_2_FC ≤ − 1 or ≥ 1. Significant upregulated and downregulated gene lists were submitted to DAVID using default settings [67] for gene enrichment analysis based on *A*. *thaliana* database annotations, to investigate functions and processes involved in the response of *H*. *lanatus* to extreme soil pH (Additional files 2, 3, 4, 5, 6, 7, 8 and 9). GO [68] terms outputted from DAVID with a Benjamini-corrected *p* value ≤ 0.01 were submitted to REViGO [69] to remove redundant GO terms using default settings. Hierarchical cluster heatmaps were generated using DESeq2 [66] and gplots [70]. A Venn diagram for root and shoot significant genelists was generated using venny [71].

Microbial transcripts showed lower expression levels compared to plant-assigned transcripts, but all samples showed similar numbers of mapped microbial-annotated reads. Therefore, all 36 samples were retained for DESeq2 [66] analysis to determine differential gene expression for microbial-annotated transcripts. A clustering heatmap for microbial transcripts was generated in R (hclust and heatmap2). DESeq2 analysis was conducted with addition of 5 to all raw counts to aid estimation of significant log_2_FCs within the generally low count microbial transcriptome data. Microbial transcripts were considered significantly differentially expressed if FDR <  0.05, absolute log_2_FC ≤ − 1 or ≥ 1, and the number of mapped reads crossed a significant expression threshold. This was set as ≥ 5 mapped reads in at least 3 samples across each of the following 4 treatment types: (a) roots grown on acid bog soil (RA, 8 samples), (b) roots grown on limestone soil (RL, 9 samples), (c) shoots grown in acid bog soil (SA, 10 samples) and (d) shoots grown in limestone soil (SL, 9 samples) to aid identification of treatment effects. This enabled transcripts that passed these thresholds to be deemed as significantly expressed in the RA, RL, SA, SL transcriptome profiles, and was incorporated in response to low microbial read counts. Tables, piecharts and a Venn diagram [71] were subsequently generated to compare the number of significantly expressed transcripts in these four treatment groups. Within each of the four treatment groups (RA, RL, SA, SL), the number of microbial transcripts showing a significant ecotype effect was recorded for various taxonomic designations. Principal component analysis was performed in R with vegan [72] on expressed root and shoot transcripts of plants and the eukaryotic microbiome. Variance partition analysis was performed in R with variancePartition [73] on root expressed transcripts of plant, the eukaryotic microbiome and *Phialocephala*.

### Quantitative real-time PCR (qPCR)

The 19 shoot samples were used for qPCR to verify RNA-Seq gene expression calls using primers for four target genes and primers for 18S [74] as an endogenous control. cDNA and a reverse transcription (RT) control were produced using a QuantiTect Reverse Transcription Kit (QIAGEN), incorporating a DNA removal step. qPCR reactions, no template controls and RT controls, were conducted in triplicate using 10 μl PrecisionPlus SYBRgreen Mastermix (Primerdesign, UK), 200 nM per primer and 1 μl cDNA or deionized water in a 20 μl reaction. Reactions were conducted using a realplex Mastercycler epgradient S (Eppendorf, Germany), and standard curve data was used to calculate reaction efficiencies for all primer pairs. Melt curves were employed to check for non-specific amplification and contamination. Expression was normalized to 18S, and statistical analyses were conducted using GLMs and post hoc Tukey tests in Minitab. Where there was non-normality, log_2_-transformed data was used. Pair-wise fold changes and standard errors plus log_2_FCs were calculated from the mean normalized expression levels for each treatment, and regressions of RNA-Seq log_2_FC against qRT-PCR log_2_FC were conducted in SigmaPlot 2001.

## Results

### Physiochemical analysis of the soils

LoI showed the *A* soil to be primarily organic (LoI 97.2%) and *L* soil minerogenic (LoI 5.8%) (Additional file 2). The *L* soil, primarily composed of decomposed substrate, is a clay marl. The organic versus minerogenic nature of these soils is illustrated by their mineral content, where the content of every mineral element was much higher in the mineral versus organic soil, including typical soil markers such as titanium and aluminum (Additional file 2).

### Shoot elemental content

There was greater accumulation of As, Cu, K and Rb, and lower Ni, in *a* than in *l* (Table 1). Furthermore, accumulation of K and Rb was greater in *a* than *l* in both soils, but this was more marked on *A*, as indicated by significant soil and ecotype interaction effects. Accumulation of P, Mg, As and Rb was significantly greater in plants grown on *A*, compared to *L*.

**Table 1:** Shoot mineral contents of reciprocally transplanted *H*. *lanatus* shoots as obtained using ICP-MS

	*p* value			Mean content (ppm)				Standard error (ppm)			
Element	Soil effect	Ecotype effect	Interaction	*Aa*	*Al*	*La*	*Ll*	*Aa*	*Al*	*La*	*Ll*
Arsenic	< 0.001	< 0.01	N.S.	1.9	0.76	0.37	0.09	0.63	0.16	0.13	0.013
Copper	N.S.	< 0.01	N.S.	12	9	11	9	0.35	0.76	1.2	0.98
Potassium	N.S.	< 0.01	< 0.05	64,000	17,000	49,000	40,000	7900	11,000	4000	10,000
Magnesium	< 0.05	N.S.	N.S.	3200	2800	2700	2200	290	240	210	7.3
Nickel	0.137	< 0.05	N.S.	2.1	4.4	3.6	6.1	0.56	1.2	0.74	1.3
Phosphorus	< 0.05	N.S.	N.S.	7400	6600	2900	5000	1400	760	330	1200
Rubidium	< 0.001	< 0.01	< 0.01	42	24	13	12	0.82	3.4	2.1	0.98

*A* acid bog soil, *L* limestone quarry soil, *a* acid bog plant ecotype, *l* limestone quarry plant ecotype

### Meta-transcriptome assembly

The sequential transcriptome assembly and annotation resulted in 108,335 transcripts, of which 31,098 were annotated as plant and 77,237 as non-plant, to which each sample from the reciprocal transplant experiment aligned. After read alignment and filtering to remove lowly expressed genes, 34,906 transcripts remained, of which 22,487 were assigned as plant and 12,419 as non-plant. Retained non-plant transcripts were re-annotated based on the best score against nr or fungal/protist databases, resulting in 7716 assigned as fungi, 1141 as protist (Oomycetes) and 2254 as protist (other), while 251 transcripts were re-assigned as nematodes (Additional file 11). Nematode transcripts and those not assigned (1057) to any of these groups were removed from further analyses. For assigned transcripts, KOG annotations were obtained for 16,739 plant, 6813 fungal, 1073 protist (Oomycete) and 2107 protist (other) annotated transcripts (Additional file 3).

### Plant gene expression and functional analysis

**Fig. 1:**
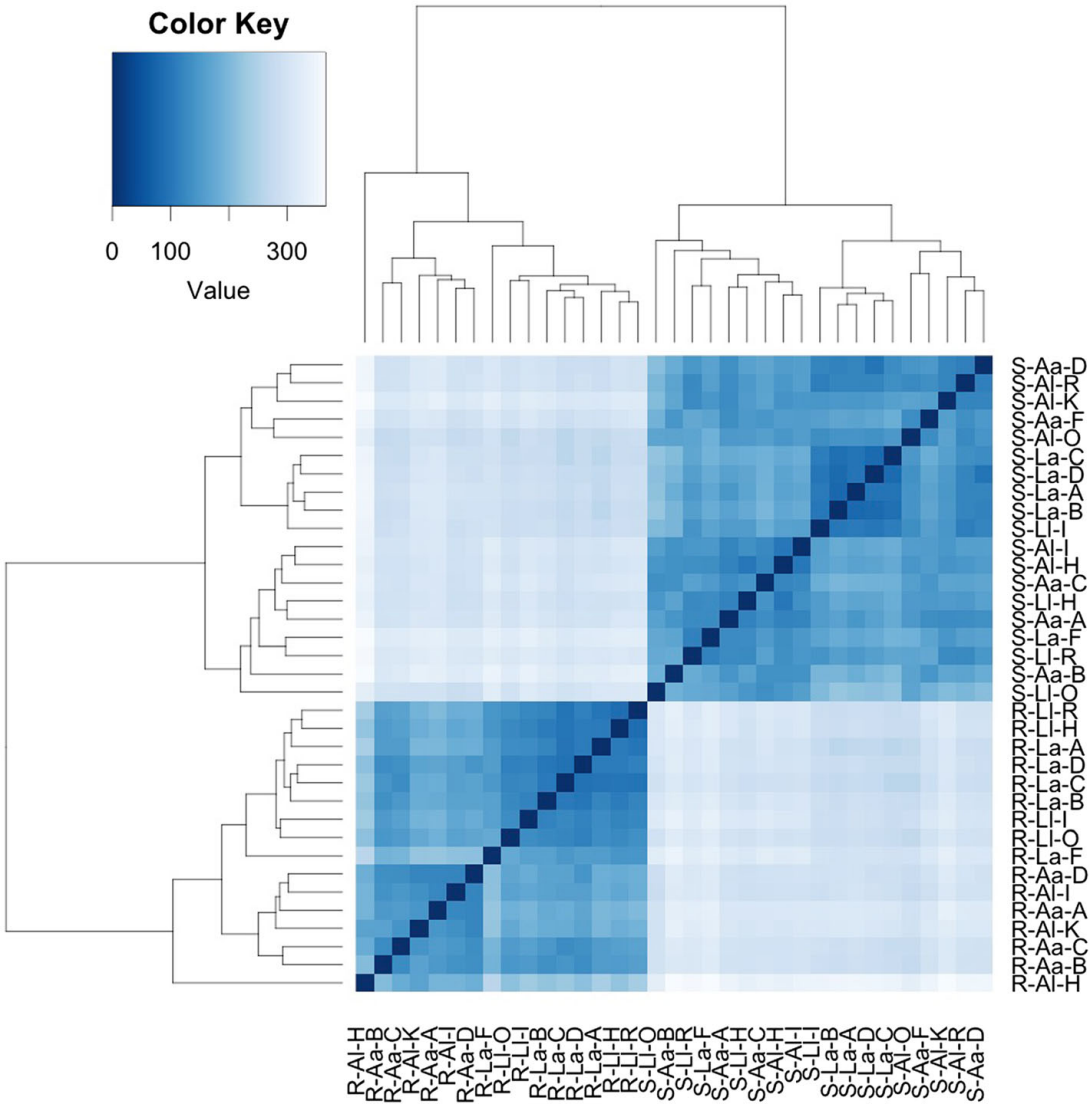
Hierarchical cluster heatmap of *H*. *lanatus* plant-annotated transcripts, generated using normalized gene counts with DESeq2 and gplots in R. The first letter refers to plant (S shoot, R root), the second to soil type (*A* acid bog soil, *L* limestone quarry soil), the third to plant ecotype (*a* acid bog plant ecotype, *l* limestone quarry plant ecotype) and the fourth to the individual plant ID (acid bog plant IDs A, B, C, D, F; limestone quarry plant IDs I, H, R, O, K)

**Fig. 2:**
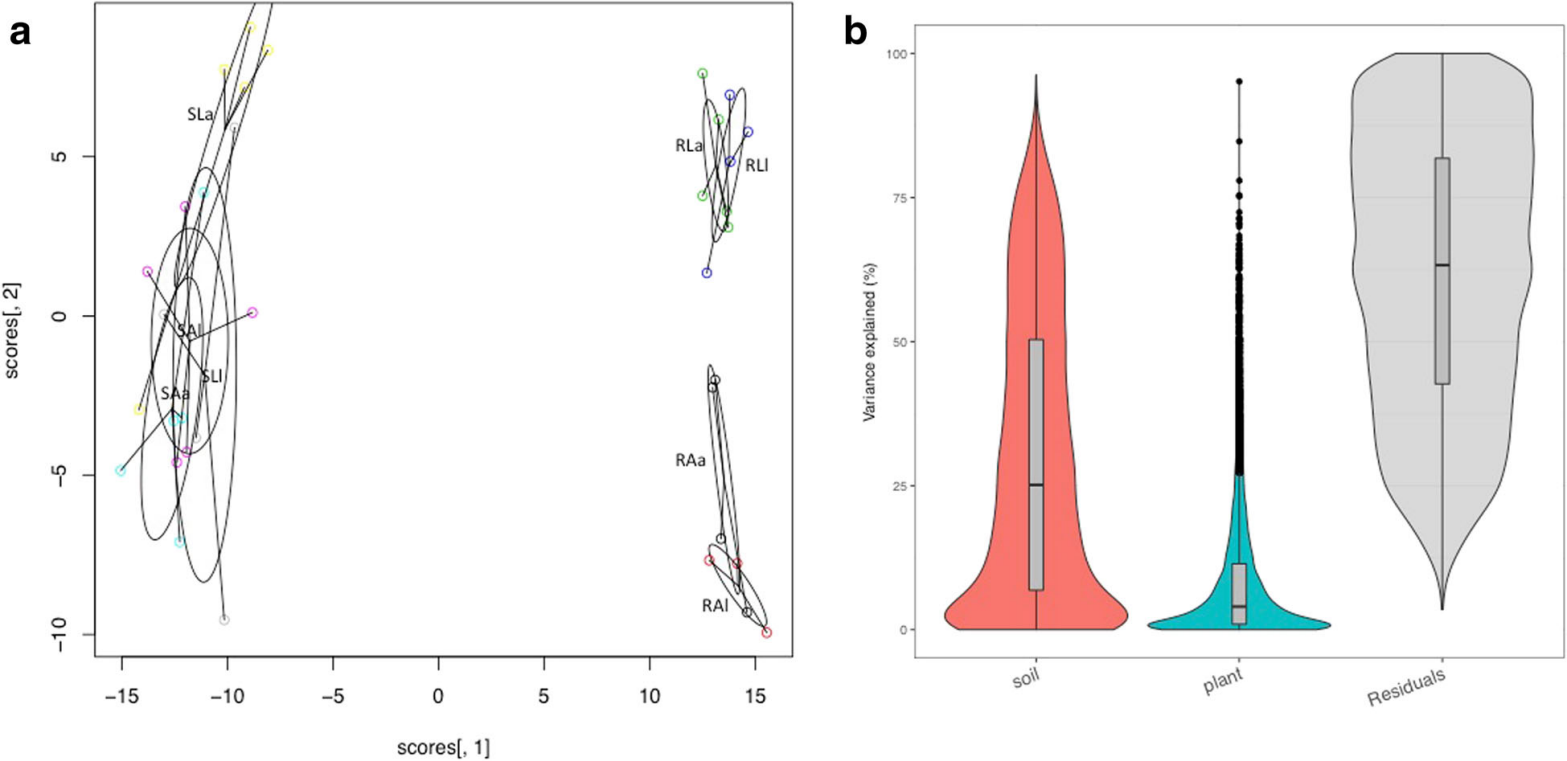
**a** PCoA analysis plot of shoot and root *H*. *lanatus* plant transcriptome data generated using the vegan package in R. SAa shoot acid bog soil, acid plant; SAl shoot acid bog soil, limestone plant; SLa shoot limestone soil, acid plant; SLl shoot limestone soil, limestone plant. RAa root acid bog soil, acid plant; RAl root acid bog soil, limestone plant; RLa root limestone soil, acid plant; RLl root limestone soil, limestone plant. **b** Violin plot showing the contributions of soil type, plant ecotype and residuals to variation in the plant root gene expression data. Generated using the variancePartition package in R

The hierarchical cluster heatmap (Fig. 1) and PCoA plot (Fig. 2a) of plant-assigned gene expression showed clear separation of root and shoot samples, with soil type separation evident within root samples, but not in shoots. For shoots and roots, 4 of 5 *a* grown on *L* clustered together, indicating a strong consistency of gene expression (Fig. 1). Soil effect (*L*, *A*) in roots accounted for ~ 25% of gene expression variation, while the plant ecotype effect (*l*, *a*) accounted for ~ 4% (Fig. 2b).

**Fig. 3:**
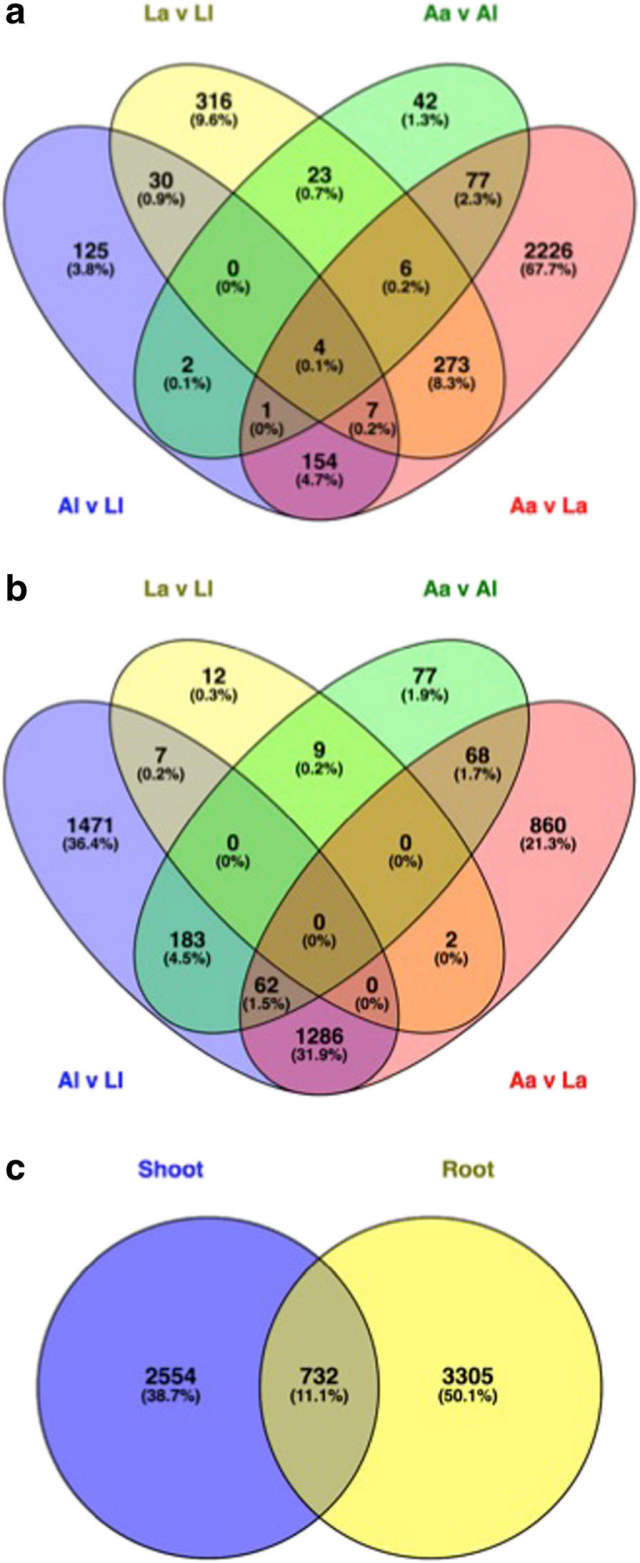
Venn diagram of significantly differentially expressed *Holcus lanatus* plant-annotated transcripts. Significance determined as FDR ≤ 0.05, recalculated absolute log_2_FC ≥ 1 or ≤ − 1, BLAST report ≥ 70% identity and ≥ 70% coverage. **a** Plant transcripts in shoot. **b** Plant transcripts in root. **c** Comparison of significantly differentially expressed transcripts obtained for shoots and roots. *L* limestone quarry soil, *A* acid bog soil, *a* acid bog plant ecotype, *l* limestone quarry plant ecotype; pairwise comparisons *La v Ll* ecotype effect on limestone soil, Aa v Al ecotype effect in acid bog soil, Al v Ll soil effect in limestone plant, Aa v La soil effect in acid plant

Of the 22,487 plant-assigned genes, 6591 were differentially expressed in at least one pairwise comparison, with fewer differentially expressed genes (DEGs) found in shoots (3286 DEGs) (Fig. 3a) than in roots (4037 DEGs) (Fig. 3b), with overlap of 732 DEGs (Fig. 3c). The effect of soil type on differential gene expression was consistently greater than that of plant ecotype, for both shoots (Fig. 3a) and roots (Fig. 3b); a total of 2905 soil effect vs. 781 ecotype effect DEGs were identified in shoots and 3939 soil effect vs. 420 ecotype effect DEGs in roots (Fig. 3). This trend was also reflected in the enriched GO terms obtained for each pairwise comparison in roots and shoots (Additional files 4 and 5).

There were marked differences in soil type response between *a* and *l* ecotypes. In shoots, *a* showed a greater response to soil type than *l* (2748 vs. 323 DEGs) (Fig. 3a). The opposite was true in roots, where *l* showed a greater response to soil type compared to *a* (3009 vs. 2278 DEGs) (Fig. 3b). There were many overlapping soil responses for *a* and *l* for roots (1348 DEGs), indicating common root responses to soil type in both ecotypes (Fig. 3b); this was less pronounced in shoots where only 166 DEGs overlapped between *a* and *l* (Fig. 3a).

The shoot soil type response for *a* involved GO terms cell wall and responses to stressors including salt, cadmium, toxic substances, bacteria and wounding (Tables 2 and 3, Additional files 4 and 5). A number of stress response and transport-associated genes were identified as upregulated in *a* shoots on *A*, compared to *L*, including cation-H+ antiporter 19, K transporter 16, K transporter 1 and nitrate transporter 1.5, with reported function in NO_3_^−^ dependent K translocation (Additional file 6, references in Additional file 7). Genes GO-annotated as involved in stress response included cinnamate beta-D-glucosyltransferase, involved in phytochelatin production and conversion of xenobiotic substances, and cadmium/zinc-transporting ATPase HMA1, involved in cation transport, particularly of Cu (Additional file 6). Also upregulated were genes involved in pathogen defense, including plasma membrane leucine-rich repeat receptor kinase 2 (PEPR2), involved in detecting fungal effectors to initiate plant defenses (Additional file 6). Shoots of *a* on *A* compared to *L* also upregulated a transcript annotated as hydroxycinnamoyl-coenzyme A shikimate, reported to affect lignin composition (Additional file 6). Furthermore, 12-oxophytodienoate reductase 1 and 12-oxophytodienoate reductase 7, genes reported to be involved in jasmonic acid (JA) biosynthesis, were upregulated in *a* shoots on *A*, compared to *L* (Additional file 6), with (hemi)biotrophic fungi known to manipulate JA to enable colonization. Response of *l* shoots to soil was more limited, with fewer DEGs and enriched GO terms. Response to wounding stress was upregulated in *A* soil, and membrane-associated genes were enriched in *L* soil (Tables 2 and 3).

**Table 2:** Selected significantly enriched plant-assigned GO terms obtained from DAVID, for various shoot and root pairwise comparisons

Classification	GO ID		GO description	*p* value (Benjamini)	FE
Shoot *Aa v La* upregulated DEGs					
Stress	BP	GO:0009651	Response to salt stress	1.54E−03	2.03
	BP	GO:0046686	Response to cadmium ion	1.67E−03	2.26
	BP	GO:0009636	Response to toxic substance	5.81E−06	6.15
	BP	GO:0009611	Response to wounding	3.65E−08	3.89
	BP	GO:0042742	Defense response to bacterium	5.51E−03	2.29
Shoot *Aa v La* downregulated DEGs					
Cell wall	CC	GO:0005618	Cell wall	1.32E−03	1.97
Shoot *Al v Ll* upregulated DEGs					
Stress	BP	GO:0009611	Response to wounding	3.71E−06	10.62
Shoot *La v Ll* downregulated DEGs					
Stress	BP	GO:0009407	Toxin catabolic process	5.20E−05	13.36
	BP	GO:0009636	Response to toxic substance	1.44E−03	8.95
	BP	GO:0042742	Defense response to bacterium	2.51E−03	3.71
Root *Aa v La* upregulated DEGs					
Stress	BP	GO:0009651	Response to salt stress	8.43E−04	2.43
	BP	GO:0046686	Response to cadmium ion	1.04E−04	3.02
Root *Aa v La* downregulated DEGs					
Stress	BP	GO:0009651	Response to salt stress	9.08E−04	2.17
	BP	GO:0046686	response to cadmium ion	1.79E−03	2.30
Phosphate	MF	GO:0003993	Acid phosphatase activity	9.00E−05	6.10
	BP	GO:0006817	Phosphate ion transport	6.02E−03	7.06
	MF	GO:0015114	Phosphate ion transmembrane transporter activity	2.07E−03	10.57
	CC	GO:0009505	Plant-type cell wall	1.48E−09	3.33
	BP	GO:0071555	Cell wall organization	4.56E−03	2.28
Nitrate	BP	GO:0015706	Nitrate transport	7.42E−03	8.34
Lignin	BP	GO:0009809	Lignin biosynthetic process	1.85E−03	4.56
Root *Al v Ll* upregulated DEGs					
Stress	BP	GO:0009651	Response to salt stress	4.14E−04	2.64
	BP	GO:0046686	Response to cadmium ion	3.73E−06	3.50
	BP	GO:0009636	Response to toxic substance	9.75E−03	5.88
Cell wall	CC	GO:0005618	Cell wall	3.51E−09	3.30
Root *Al v Ll* downregulated DEGs					
Phosphate	MF	GO:0003993	Acid phosphatase activity	1.10E−03	4.23
	CC	GO:0009505	Plant-type cell wall	1.58E−21	3.97
	BP	GO:0071555	Cell wall organization	1.23E−13	3.29
	BP	GO:0009834	Plant-type secondary cell wall biogenesis	1.59E−04	5.22
	BP	GO:0010411	Xyloglucan metabolic process	1.13E−04	5.47
	BP	GO:0045492	Xylan biosynthetic process	9.33E−03	5.47
	BP	GO:0045493	Xylan catabolic process	6.73E−04	11.49
Nitrate	MF	GO:0015112	Nitrate transmembrane transporter activity	8.80E−03	7.34
Lignin	BP	GO:0009809	Lignin biosynthetic process	1.09E−07	5.31
	BP	GO:0046274	Lignin catabolic process	3.95E−04	8.21
Iron	MF	GO:0020037	Heme binding	5.41E−04	2.00
Root *Aa v Al* upregulated DEGS					
Cell wall	BP	GO:0009834	Plant-type secondary cell wall biogenesis	4.23E−05	19.79
	CC	GO:0009505	Plant-type cell wall	2.03E−04	4.80
Lignin	BP	GO:0009809	Lignin biosynthetic process	3.79E−03	11.20

*A* = acid bog soil, *L* = limestone quarry soil, *a* = acid bog plant ecotype, *l* = limestone quarry plant ecotype. GO categories: BP = biological process; CC = cellular component; MF = molecular function. FE = fold enrichment. The reported p-value is the Benjamini *p* value, corrected for multiple testing

**Table 3:** Summarized overview of enriched GO terms based on complete REViGO simplification results, following DAVID GO analysis, for all shoot and root pairwise comparisons

	Classification, based on REViGO output, using similarity = 0.7	Shoot				Root			
		*Aa v La*	*Al v Ll*	*Aa v Al*	*La v Ll*	*Aa v La*	*Al v Ll*	*Aa v Al*	*La v Ll*
Upregulated DEGs	Membrane	x		x	x	x	x	x	
	Cell wall					x	x	x	
	Binding	x							
	Signal transduction	x		x					
	Transport	x							
	Post-translational modification	x		x					
	DNA replication/gene expression						x		
	Response to stimulus and stress	x	x			x	x		
	Phosphate starvation and acquisition								
	Lignin production and biosynthesis							x	
	Oxidation-reduction/anti-oxidation	x						x	
	Cell division and growth								
	Microtubule motor/assembly						x		
	Glutathione	x							
	Hormones	x					x		
	Epigenetics								
	Cellulose production								
Downregulated DEGs	Membrane	x	x		x	x	x	x	
	Cell wall	x				x	x		
	Binding	x			x	x	x		
	Signal transduction	x			x		x		
	Transport				x	x	x		
	Post-translational modification				x				
	DNA replication/gene expression	x							
	Response to stimulus and stress				x	x			
	Phosphate starvation and acquisition					x	x		
	Lignin production and biosynthesis					x	x		
	Oxidation-reduction/anti-oxidation					x	x		
	Cell division and growth	x							
	Microtubule motor/assembly	x				x	x	x	
	Glutathione				x				
	Hormones								
	Epigenetics	x							
	Cellulose production					x	x		

*A* acid bog soil, *L* limestone quarry soil, *a* acid bog plant ecotype, *l* limestone quarry plant ecotype

Soil type responses identified in *l* roots included signal transduction, transport, response to stimulus and stress, phosphate starvation and acquisition, lignin production and biosynthesis and oxidation-reduction amongst others (Tables 2 and 3, Additional files 4 and 5). A number of genes involved in lignin biosynthesis and composition, such as laccases 5 and 11 and cinnamyl alcohol dehydrogenase, were upregulated in *l* roots on *L*, relative to *A* (Additional file 6). Transport genes upregulated in *l* on *L*, compared to *A*, were involved in nutrient uptake and transport, particularly for N and P. These included a range of transporters that facilitate uptake of N under low N conditions, such as nitrate transporters 1.1 and 1.5, and high-affinity nitrate transporters 2.1, 2.4, 3.1 and 3.2 (Additional file 6). Nitrate transporter 1.1 is a dual-affinity nitrate transporter thought to be involved in multiple phases of nitrate uptake. With regards to P-assimilation, genes upregulated in *l* roots on *L*, compared to *A*, included purple acid phosphatases, which hydrolyse phosphomonoesters to release P and are implicated in phosphate use efficiency, as well as high-affinity K transporters, including K transporters PT1-11 and PT1-13, known to be important for AM symbiosis (Additional file 6). Also upregulated in *l* on *L* were genes involved in Fe uptake, including phytosiderophore-chelated Fe. The latter included iron-phytosiderophore transporter YSL15 (Additional file 6). A number of genes involved in amelioration of oxidative stress were upregulated in *l* on *L* compared to *A*, including numerous class III plant peroxidases, including peroxidases 1 and 70 (Additional file 6). The former is reported as a central component in the reactive oxygen gene network response, facilitating amelioration of oxidative stress, with the latter regulated by plant hormones JA and salicylic acid (SAc) in response to pathogen elicitors. Other pathogen defense genes were also upregulated in *l* on *L*, compared to *A*, including isoflavone reductase and premnaspirodiene oxygenase (Additional file 6). With respect to K transport and homeostasis, K channel AKT2 and cation/H(^+^) antiporter 15 were upregulated in *l* roots on *L*, while K transporter 18 and cation transporter HKT8 were upregulated on *A* (Additional file 6). Far fewer genes were upregulated in *l* on *A* compared to *L*, but those that were included some catalases and class III plant peroxidases, including peroxidase 70, plus the aforementioned genes involved in K homeostasis (Additional file 6).

The soil type responses identified in *a* roots were broadly similar to those observed in *l* and included transport, response to stimulus and stress, phosphate starvation and acquisition, lignin production and biosynthesis and oxidation-reduction amongst others (Tables 2 and 3, Additional files 4 and 5). As in *l*, lignin biosynthesis-related genes were upregulated in *a* on *L* compared to *A*, suggesting a role of lignification in the *L* environment in both *a* and *l*. As observed in *l*, P, N, Fe uptake and within-plant transport genes were upregulated in *a* on *L* relative to *A*, including upregulation of the same nitrate transporters as in *l*, excepting nitrate transporter 1.1, and with the addition of nitrate transporter 1.2 and high-affinity nitrate transporter 2.5, which plays a role in acquisition and remobilization in nitrogen-starved plants (Additional file 6). As in *l*, there was upregulation of genes involved in uptake of phytosiderophore-chelated Fe on *L* compared to *A*, including iron-phytosiderophore transporter YSL15 with the addition of metal-nicotianamine transporter YSL12 (Additional file 6). In both ecotypes, P transport genes were upregulated on *L* compared to *A*, including P transporters PT1-11 and PT1-13, with the addition of PT1-10 and a high-affinity K transporter in *a* roots (Additional file 6). PT1-10, PT1-11 and PT1-13 are thought to be involved in the establishment of mycorrhizal symbiosis and induced during AM colonization, with PT1-11 implicated in P acquisition via the AM symbiosis. Thus, lignification and increased investment in P, N and Fe nutrient acquisition appear to be a response to *L* in both *H*. *lanatus* ecotypes, which is reiterated by enrichment of GO terms related to these processes in roots of ecotypes in response to soil type (Table 2).

Other enriched GO terms shared by both roots of both ecotypes in response to soil type related to membrane, cell wall and oxidation-reduction (Table 3). As for *l*, *a* roots were enriched in pathogen defense-related genes such as isoflavone reductase and premnaspirodiene oxygenase, with the addition of basic endochitinase A on *L* compared to *A* (Additional file 6). Regarding genes involved in oxidation-reduction and amelioration of oxidative stress, both *a* and *l* roots upregulated a number of class III plant peroxidases in *L* compared to *A* (Additional file 6), with production of antioxidant compounds also implicated in *a*. With respect to K transport and homeostasis genes, both *a* and *l* upregulated K channel AKT2 and cation/H^+^ antiporter 15 in *L*, and upregulated K transporter 18 and cation transporter HKT8 in *A*. Additionally, *a* also upregulated K transporter 5 in *L* and K channel KOR2 in *A* (Additional file 6). As in *a* shoots, PEPR2, a gene involved in detecting fungal effectors to initiate plant defenses was upregulated in *a* roots in *A*, compared to *L*. PEPR2 was not upregulated by *l* shoots or roots in *A* (Additional file 6).

Although soil type was the overriding effect, there was also an effect of plant ecotype on differential gene expression. This was greater on *L* than on *A* in shoots (659 vs. 155 DEGs), in contrast to roots, where ecotype effect was greater on *A*, compared to *L* (399 vs. 30 DEGs) (Fig. 3). The REViGO enrichment analysis correspondingly showed a larger number of summarized enriched GO terms for ecotype effect on *L*-grown shoots compared to *A*-grown shoots, and in *A* compared to *L*-grown roots (Table 3, Additional file 5).

Differences in gene expression responses between *a* and *l* shoots on *L* involved membrane, defense response and response to toxins amongst others (Tables 2 and 3, Additional files 4 and 5). Stimulus and stress response-related genes upregulated on *L* in *l* shoots, as compared to *a*, included glutathione S-transferases, a large family with many members involved in detoxification and amelioration of oxidative stress, and pathogen defense genes such as disease resistance protein RPS2 and coronatine-insensitive protein 1, the latter known to be associated with pathogen defense and JA response (Additional file 6). Shoots of *l* on *L* also upregulated genes involved in K-uptake and homeostasis and Na^+^ accumulation, in comparison to *a*, in particular, cation-H^+^ antiporter 19, K transporter 16 and K transporter 1 (Additional file 6). Cation-H^+^ antiporter 19 is reported to be associated with K homeostasis in response to alkaline conditions, and other K transporters are reported to be involved in salt stress tolerance. In contrast, K channel KOR2, known to be involved in K^+^ release into xylem sap, was upregulated in *a* compared to *l* shoots on *L* (Additional file 6). Overall, the ecotype effect in shoots on *A* was less pronounced than on *L* (Fig. 3a), and was restricted to membrane, signal transduction and port-translational modification-associated genes (Table 3, Additional files 4 and 5).

For roots, significant ecotype responses on *A* involved the cell wall, lignin biosynthesis and oxidation-reduction related genes amongst others (Tables 2 and 3, Additional files 4 and 5). Lignin biosynthesis and composition genes, such as cinnamyl alcohol dehydrogenase, laccase 4, laccase 5 and laccase 11, were upregulated in *a*, relative to *l*, suggesting lignin usage in *a* is related to stressors associated with *A* soil, which *l* does not replicate when grown on *A* (Additional file 6). Roots of *a* displayed greater expression of a range of class III plant peroxidases compared to *l* roots in *A*, including peroxidase 1, central to amelioration of oxidative stress, and peroxidase 70, regulated by JA and SAc, and in response to pathogen elicitors (Additional file 6). Furthermore, *a* roots had higher expression of K channel AKT2 compared to *l* roots when grown on *A* (Additional file 6). This gene is known to be involved in phloem loading and unloading of K^+^. In contrast to *A*, the ecotype effect in roots on *L* was much less pronounced (Fig. 3b), involving only 30 genes with no enriched GO terms (Table 3, Additional file 4).

Full DESeq2 results and database annotations for plant annotated transcripts are shown in Additional file 8 with corresponding sequences in fasta format in Additional file 9.

### RNA-Seq gene expression verification using qPCR

The reliability of the RNA-Seq data and differential expression calls was investigated using qPCR, with genes chosen to cover a range of gene expression patterns between treatments. The qPCR results verified the RNA-Seq data well, with regressions of qPCR mean treatment log_2_FC against the mean recalculated RNA-Seq counterparts showing an *R*^2^ of 98.2%. Furthermore, where the RNA-Seq indicated a significant difference in gene expression levels between two treatments (FDR <  0.05 and recalculated log_2_FC ≤ − 1 or ≥ 1), the qPCR results corroborated this (*p* <  0.05, log_2_FC ≤ − 1 or ≥ 1) (Additional file 10). Primers used for qPCR are shown in Additional file 10.

### Microbial gene expression and functional analysis

Transcripts best annotated as eukaryotic microbes were successfully assembled and functionally annotated (Additional file 3). Fungal and protist transcripts corresponding to all four KOG groups (cellular process and signalling, information storage and processing, metabolism, poorly characterized) were obtained, with the fungal transcriptome dominated by Ascomycetes and the protist transcriptome by Oomycete-annotated genes (Additional files 3 and 12).

**Fig. 4:**
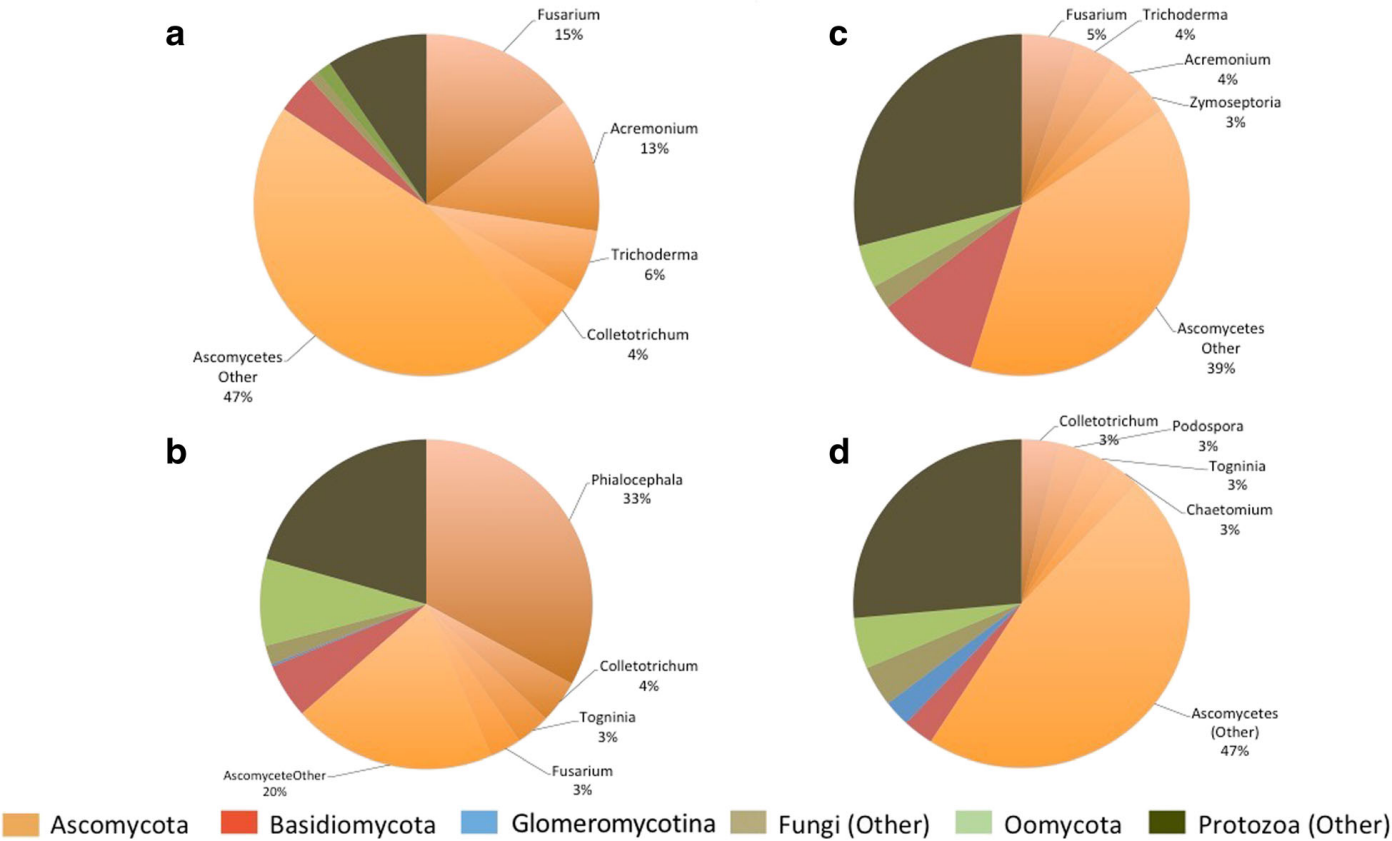
Piechart showing the relative proportion of significantly expressed transcripts for each microbial taxonomic phylum (indicated by colour) and genera (indicated by piechart labels). **a** Shoot acid bog soil, SA. **b** Root acid bog soil, RA. **c** Shoot limestone soil, SL. **d** Root limestone soil, RL. The number of significantly expressed transcripts is defined as the number of microbial-annotated transcripts that obtained ≥ 5 aligned reads in at least 3 samples in each of the treatments RA (total 8 samples), RL (total 9 samples), SA (total 10 samples), SL (total 9 samples)

**Fig. 5:**
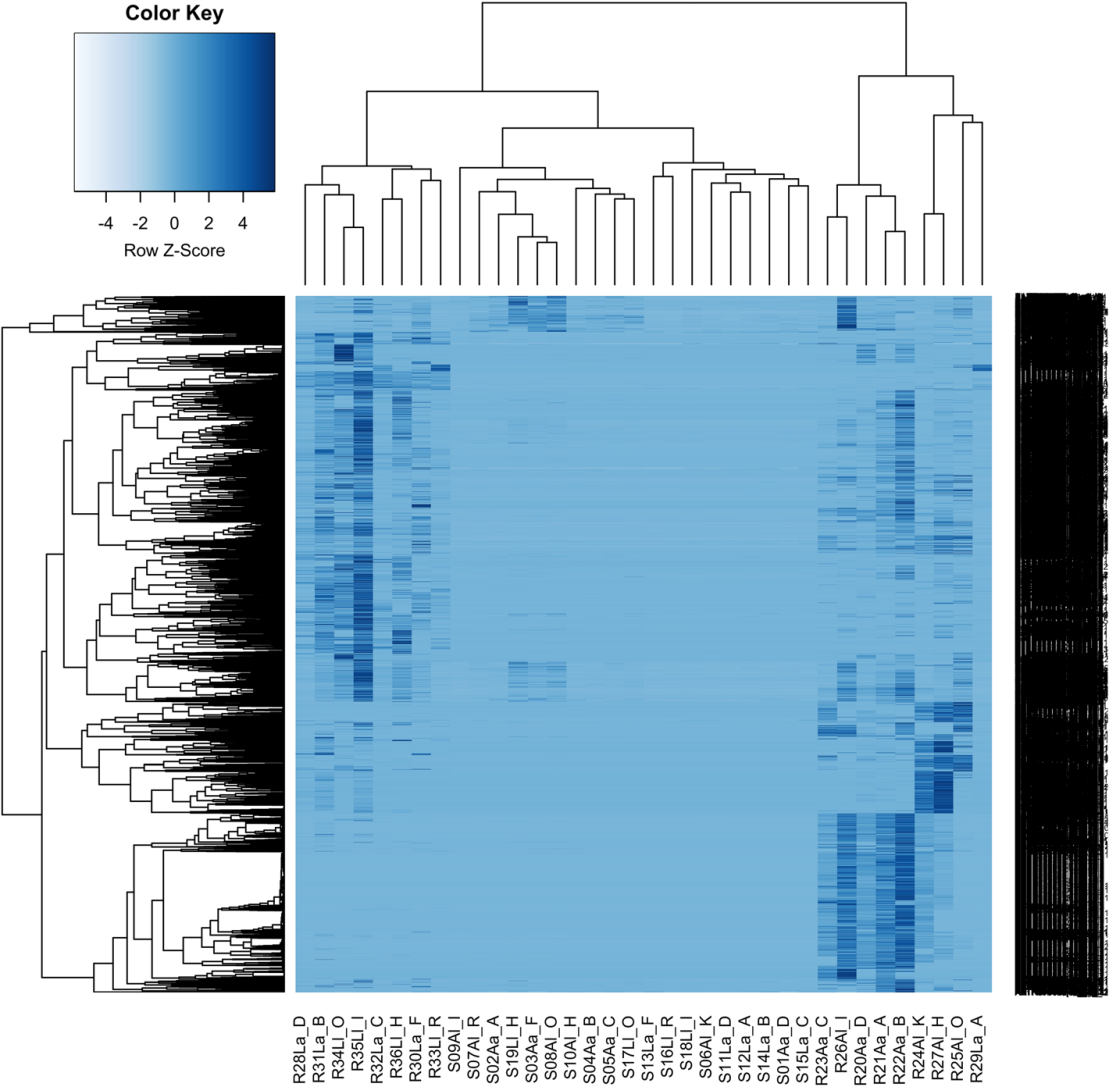
Heirarchical cluster heatmap of microbial-annotated transcripts, generated using microbial RNA-Seq count data, using hclust and heatmap2 in R. Transcripts are clustered by row, and samples by column. The first letter refers to plant (S shoot, R root), the second to soil type (*A* acid bog soil, *L* limestone quarry soil), the third to plant ecotype (*a* acid bog plant ecotype, *l* limestone quarry plant ecotype) and the fourth to the individual plant ID (acid bog plant IDs A, B, C, D, F; limestone quarry plant IDs I, H, R, O, K), the number after R or S refers to the sample number 1–36, with 1–19 being shoot samples and 20–36 root samples

**Fig. 6:**
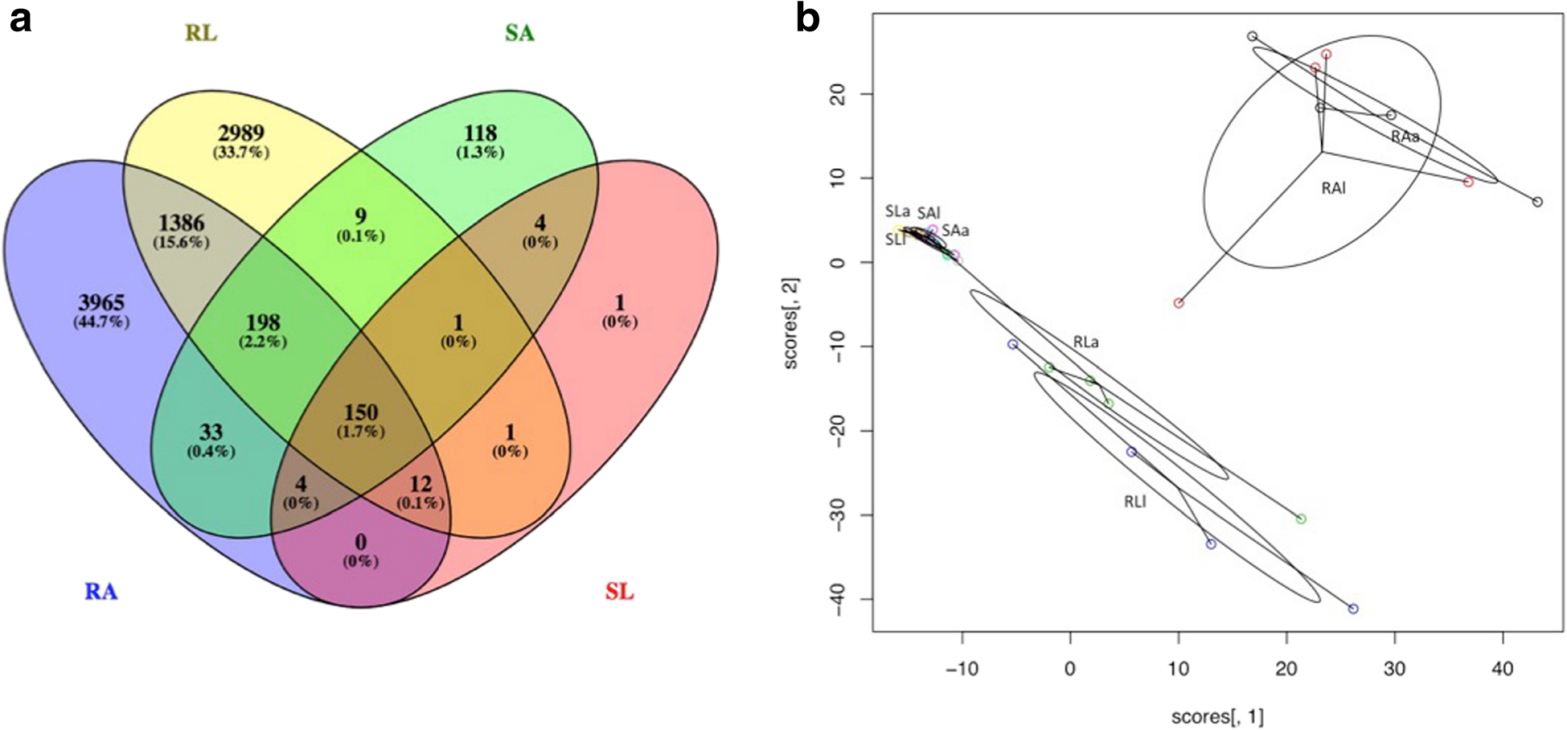
**a** Venn diagram of significantly expressed transcripts in root and shoot in each soil; root acid bog soil (RA), root limestone soil (RL), shoot acid bog soil (SA) and shoot limestone soil (SL). The number of significantly expressed transcripts is defined as the number of microbial-annotated transcripts that obtained ≥ 5 aligned reads in at least 3 samples in each of the treatments RA (total 8 samples), RL (total 9 samples), SA (total 10 samples), SL (total 9 samples). **b** PCoA analysis plot of the shoot and root microbial data generated using the vegan package in R. SAa shoot acid bog soil, acid plant; SAl shoot acid bog soil, limestone plant; SLa shoot limestone soil, acid plant; SLl shoot limestone soil, limestone plant. RAa root acid bog soil, acid plant; RAl root acid bog soil, limestone plant; RLa root limestone soil, acid plant; RLl root limestone soil, limestone plant

**Fig. 7:**
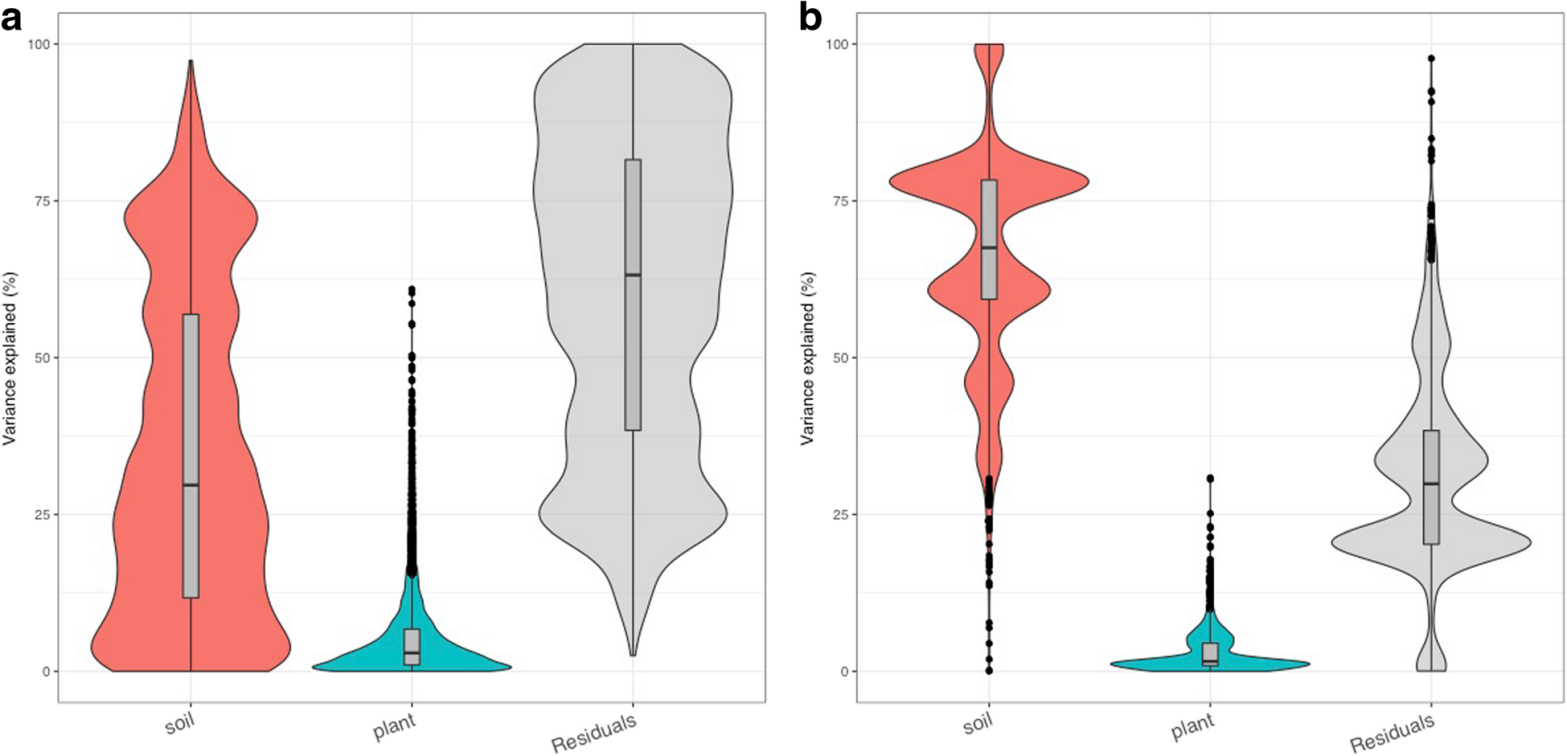
Violin plot showing the contributions of soil type, plant ecotype and residuals to variation in gene expression data of **a** all root eukaryotic microbiota and **b** root *Phialocephala* expressed transcripts. Generated using the variancePartition package in R

Fungal and protist transcripts were detected as significantly expressed in roots and shoots of *H*. *lanatus* grown on both *A* and *L* (Table 4, Fig. 4). Transcripts demonstrated a root vs. shoot effect on gene expression, as well as strong soil effects on gene expression in roots, with some evidence of a soil effect also identifiable in shoots (Figs. 5 and 6a, b). More transcripts were detected in roots than shoots, and in both, more were detected in plants grown on *A* than *L* (Table 4, Fig. 6a). Soil is accounting for more variation in root microbiome gene expression than plant ecotype, but plant ecotype also explains some of the variation (Fig. 7a).

**Table 4:** The number of significantly expressed microbial transcripts in root and shoot for each soil

	*SA* expressed at ≥ 5 reads in 3 out of 10 samples			*SL* expressed at ≥ 5 reads in 3 out of 9 samples			*RA* expressed at ≥ 5 reads in 3 out of 8 samples			*RL* expressed at ≥ 5 reads in 3 out of 9 samples		
	All	Plant effect		All	Plant effect		All	Plant effect		All	Plant effect	
		Up in *a*	up in *l*		Up in *a*	Up in *l*		Up in *a*	Up in *l*		Up in *a*	Up in *l*
Protists (Oomycetes)	7	0	0	7	0	0	490	8	365	239	0	12
Protists (other)	49	0	0	50	0	0	1183	68	443	1254	19	80
Ascomycetes	437	0	0	95	2	4	3646	421	166	2804	20	1139
Basidiomycetes	19	0	0	17	0	0	309	8	62	139	1	6
Glomeromycotina	1	0	0	1	0	0	12	1	2	122	0	1
Fungi (other)	4	0	0	3	0	0	108	5	31	188	0	10
Ascomycota (selected)												
*Phialocephala*	3	0	0	0	0	0	1886	289	3	21	0	4
*Colletotrichum*	22	0	0	2	0	0	63	2	8	166	2	65
*Fusarium*	76	0	0	9	0	3	105	1	24	104	0	25
*Acremonium*	66	0	0	6	0	0	47	1	10	39	0	11
*Trichoderma*	31	0	0	7	0	0	58	5	8	66	0	22

The number of significantly expressed microbial transcripts (All) is defined as the number of transcripts that obtained ≥ 5 aligned reads in at least three samples from each of the following treatments: root acid bog soil (RA), root lime-stone soil (RL), shoot acid bog soil (SA) and shoot lime-stone soil (SL). Significant ecotype effects (absolute log_2_FC ≤ −1 or ≥ 1, FDR < 0.05) as identified by DESeq2 analysis are reported in subsequent columns for each treatment group under the heading plant effect

Fungal-annotated transcripts were more prevalent than protist-annotated transcripts in roots and shoots on both soils, with most detected fungal transcripts best annotated as non-AM fungi and mostly assigned to the Ascomycota (Table 4, Fig. 4). Furthermore, greater numbers of Ascomycete-annotated genes were significantly expressed in roots than shoots, but in both, more were detected in plants grown on *A* than *L* (Table 4). Ascomycota-annotated genes significantly expressed in roots grown on *A* were dominated by the genus *Phialocephala*, with many of these showing upregulation in *a*, compared to *l* roots (Table 4, Fig. 4b). In contrast, there was no single dominant Ascomycete genus significantly expressed in roots or shoots on *L*, although *Colletotrichum*-annotated transcripts were most numerous in roots and *Fusarium*-annotated transcripts in shoots (Table 4, Fig. 4). These genera, plus others including *Acremonium* and *Trichoderma* showed higher expression levels in roots of *l* compared to *a*, in both soils, particularly *L* (Table 4).

While most non-AM-assembled transcripts were annotated as Ascomycota, transcripts identified as other fungal phyla including Basidiomycota, Chytridiomycota, Cryptomycota and Zygomycota were identified, with greater prevalence in roots compared to shoots (Table 4, Fig. 4, Additional file 12). Basidiomycetes showed greater activity from *A*-grown roots than *L*, and within *A*, more Basidiomycete-annotated transcripts were upregulated in *l* roots than *a* (Table 4). Basidiomycete-annotated transcripts upregulated in *l* roots compared to *a* roots in *A* soil were predominantly annotated as KOG translation and energy production (Additional file 13).

Most Ascomycota transcripts significantly expressed at a level of ≥ 5 counts in ≥ 3 samples in roots from *A* were KOG annotated as involved in metabolism, including energy production and conversion and transport and metabolism of carbohydrates, amino acids and lipids (Table 5). Roots from *L* were mainly KOG annotated as information storage and processing, particularly translation, ribosomal structure and biogenesis. This was also the case for shoots from *A* and *L*, although transcripts KOG annotated as metabolism were also well represented in shoots from *A*, and cellular processes and signalling in shoots from *L*. In all categories, fewer Ascomycota transcripts were annotated in shoots, due to lower overall transcript detection compared to roots (Tables 4 and 5).

**Table 5:** Significantly expressed KOG-annotated Ascomycota transcripts in root and shoot in each soil

	*SA* expressed at ≥ 5 reads in 3 out of 10 samples			*SL* expressed at ≥ 5 reads in 3 out of 9 samples			*RA* expressed at ≥ 5 reads in 3 out of 8 samples			*RL* expressed at ≥ 5 reads in 3 out of 9 samples		
Ascomycetes	All	Plant effect		All	Plant effect		All	Plant effect		All	Plant effect	
		Up in *a*	Up in *l*		Up in *a*	Up in *l*		Up in *a*	Up in *l*		Up in *a*	Up in *l*
Cellular processes and signaling	71	0	0	28	2	0	769	89	30	729	6	274
Cell motility	0	0	0	0	0	0	1	0	0	0	0	0
Cell wall/membrane/envelope biogenesis	1	0	0	0	0	0	36	2	0	45	0	21
Cytoskeleton	7	0	0	7	2	0	96	13	7	74	1	19
Defense mechanisms	2	0	0	0	0	0	13	0	0	8	0	1
Extracellular structures	4	0	0	0	0	0	5	0	0	3	0	0
Intracellular trafficking, secretion, and vesicular transport	0	0	0	2	0	0	114	15	3	77	0	28
Nuclear structure	0	0	0	0	0	0	9	1	0	7	0	4
Posttranslational modification, protein turnover, chaperones	50	0	0	17	0	0	334	30	14	355	3	128
Signal transduction mechanisms	7	0	0	2	0	0	161	28	6	160	2	73
Information storage and processing	137	0	0	35	0	0	918	51	57	785	4	348
Chromatin structure and dynamics	7	0	0	4	0	0	40	2	2	35	0	22
Replication, recombination and repair	1	0	0	0	0	0	46	1	0	1	0	0
RNA processing and modification	1	0	0	0	0	0	128	13	2	34	1	14
Transcription	3	0	0	31	0	0	90	8	2	61	0	28
Translation, ribosomal structure and biogenesis	125	0	0	0	0	0	614	27	51	654	3	284
Metabolism	134	0	0	16	0	4	1083	113	50	763	3	319
Amino acid transport and metabolism	10	0	0	0	0	0	149	26	1	97	0	45
Carbohydrate transport and metabolism	16	0	0	4	0	0	179	32	7	162	2	91
Cell cycle control, cell division, chromosome partitioning	0	0	0	0	0	0	77	3	1	42	0	17
Coenzyme transport and metabolism	1	0	0	1	0	0	38	1	2	27	0	4
Energy production and conversion	65	0	0	8	0	4	309	19	35	267	0	88
Inorganic ion transport and metabolism	13	0	0	0	0	0	66	8	0	61	0	23
Lipid transport and metabolism	11	0	0	1	0	0	125	12	0	51	0	26
Nucleotide transport and metabolism	1	0	0	0	0	0	42	2	0	20	0	9
Secondary metabolites biosynthesis, transport and catabolism	17	0	0	2	0	0	98	10	4	36	1	16
Poorly characterized	31	0	0	1	0	0	434	67	8	253	0	96
Function unknown	2	0	0	0	0	0	85	6	0	13	0	4
General function prediction only	29	0	0	1	0	0	349	61	8	240	0	92
Total KOG annotated, expressed	373	0	0	80	2	3	3204	320	145	2528	13	1037

The number of significantly expressed KOG-annotated Ascomycota transcripts (All) is defined as the number of transcripts that obtained ≥ 5 aligned reads in at least 3 samples from each of the following treatments: root acid bog soil (RA), root limestone soil (RL), shoot acid bog soil (SA) and shoot limestone soil (SL). Significant ecotype effects (absolute log_2_FC ≤ − 1 or ≥ 1, FDR <  0.05) as identified by DESeq2 analysis are reported in subsequent columns for each treatment group under the heading plant effect

Other Ascomycota-annotated KOGs of interest included intracellular trafficking, inorganic ion transport and metabolism, signal transduction mechanisms and replication and recombination and repair. Some of these are putatively involved in fungal virulence and infectivity, with others likely to be involved in nutrient acquisition, uptake and homeostasis, likely with functions related to stresses associated with *A* or *L*. For roots grown on *A*, many significantly expressed transcripts annotated with these KOG functions and putative uses were also annotated as *Phialocephala* (Additional file 11, references in Additional file 7). A total of 225 *A*-grown root *Phialocephala* genes were upregulated in *a* compared to *l*, with 24 of these KOG annotated as amino acid transport and metabolism (Table 6), including arginase, involved in amino acid breakdown to release N (Additional file 11). *Phialocephala*-annotated genes involved in metal(loid) homeostasis, P transport, and stress tolerance were more numerous in roots grown on *A* compared to *L* (Additional file 11). Furthermore, a number of these transcripts were upregulated in roots of *a*, compared to *l*, on *A*, including a Zn transporter, inorganic P transporters, a K^+^/H^+^-antiporter and aldehyde dehydrogenases, with many of the latter upregulated in both ecotype roots when grown on *A* compared to *L*; aldehyde dehydrogenases are associated with energy production and oxidative stress tolerance. Transcripts annotated as these were also expressed by Ascomycete genera other than *Phialocephala* on both soils, with a significant number of transcripts in *l* roots showing increased expression compared to *a* in both soils (Additional file 11). A greater number of Ascomycota genes involved in stress tolerance and repair were detected in *A* than *L*, including those involved in oxidative stress response and DNA damage detection and repair, many of which were best annotated as *Phialocephala*. Fungal virulence and infectivity related genes, particularly casein kinases, transport protein Sec61 and GTP-binding ADP-ribosylation factor Arf1, were expressed in both *A* and *L* and annotated by a range of Ascomycete genera. Many of these, particularly those annotated as *Phialocephala*, were more strongly expressed in *A* (Additional file 11). Casein kinases are known to be essential for cell integrity and fungal virulence and Arf1 may be involved in fungal morphogenesis and virulence. Roots grown on *L* showed a greater number of Ascomycota-annotated ferric reductases, known to be involved in Fe acquisition than those on *A*. Of these, eight genes, including two annotated as *Colletotrichum*, were upregulated in roots of *l*, compared to *a*, on *L* (Additional file 11). Ascomycete-annotated genes from a range of genera proposed to be involved in fungal K homeostasis were detected in both soils, with a *Phialocephala*-annotated K^+^/H^+^-antiporter and Na^+^/K^+^ transporter showing greater expression on *A*, while those upregulated in *L* roots were annotated with a range of other Ascomycota genera, including a *Colletotrichum*-annotated K^+^/H^+^-antiporter. K^+^/H^+^-antiporters influence the plasma membrane potential of fungi, thereby increasing pH tolerance.

**Table 6:** Significantly expressed KOG annotated *Phialocephala* transcripts in root and shoot in each soil

	*SA* expressed at ≥ 5 reads in 3 out of 10 samples			*SL* expressed at ≥ 5 reads in 3 out of 9 samples			*RA* expressed at ≥ 5 reads in 3 out of 8 samples			*RL* expressed at ≥ 5 reads in 3 out of 9 samples		
*Phialocephala*	All	Plant effect		All	Plant effect		All	Plant effect		All	Plant effect	
		Up in *a*	Up in *l*		Up in *a*	Up in *l*		Up in *a*	Up in *l*		Up in *a*	Up in *l*
Cellular processes and signaling	1	0	0	0	0	0	370	61	0	6	0	2
Cell motility	0	0	0	0	0	0	1	0	0	0	0	0
Cell wall/membrane/envelope biogenesis	0	0	0	0	0	0	23	1	0	0	0	0
Cytoskeleton	0	0	0	0	0	0	37	6	0	0	0	0
Defense mechanisms	0	0	0	0	0	0	10	0	0	0	0	0
Extracellular structures	0	0	0	0	0	0	5	0	0	0	0	0
Intracellular trafficking, secretion, and vesicular transport	0	0	0	0	0	0	73	14	0	1	0	0
Nuclear structure	0	0	0	0	0	0	6	1	0	0	0	0
Posttranslational modification, protein turnover, chaperones	1	0	0	0	0	0	114	18	0	5	0	2
Signal transduction mechanisms	0	0	0	0	0	0	101	21	0	0	0	0
Information storage and processing	1	0	0	0	0	0	322	35	0	8	0	2
Chromatin structure and dynamics	0	0	0	0	0	0	18	1	0	0	0	0
Replication, recombination and repair	0	0	0	0	0	0	43	1	0	0	0	0
RNA processing and modification	0	0	0	0	0	0	86	10	0	0	0	0
Transcription	0	0	0	0	0	0	64	7	0	0	0	0
Translation, ribosomal structure and biogenesis	1	0	0	0	0	0	111	16	0	8	0	2
Metabolism	1	0	0	0	0	0	601	81	2	4	0	0
Amino acid transport and metabolism	0	0	0	0	0	0	117	24	0	1	0	0
Carbohydrate transport and metabolism	0	0	0	0	0	0	91	19	0	0	0	0
Cell cycle control, cell division, chromosome partitioning	0	0	0	0	0	0	46	2	0	0	0	0
Coenzyme transport and metabolism	0	0	0	0	0	0	25	1	0	0	0	0
Energy production and conversion	1	0	0	0	0	0	93	11	1	1	0	0
Inorganic ion transport and metabolism	0	0	0	0	0	0	38	7	0	0	0	0
Lipid transport and metabolism	0	0	0	0	0	0	85	6	0	0	0	0
Nucleotide transport and metabolism	0	0	0	0	0	0	33	2	0	1	0	0
Secondary metabolites biosynthesis, transport and catabolism	0	0	0	0	0	0	73	9	1	1	0	0
Poorly characterized	0	0	0	0	0	0	319	48	1	0	0	1
Function unknown	0	0	0	0	0	0	78	5	0	0	0	0
General function prediction only	0	0	0	0	0	0	241	43	1	0	0	1
Total KOG annotated	3	0	0	0	0	0	1612	225	3	18	0	5

The number of significantly expressed KOG-annotated *Phialocephala* transcripts (All) is defined as the number of transcripts that obtained ≥ 5 aligned reads in at least 3 samples from each of the following treatments: root acid bog soil (RA), root limestone soil (RL), shoot acid bog soil (SA) and shoot limestone soil (SL). Significant ecotype effects (absolute log_2_FC ≤ − 1 or ≥ 1, FDR <  0.05) as identified by DESeq2 analysis are reported in subsequent columns for each treatment group under the heading plant effect

In roots on *A*, 1612 of 3204 significantly expressed genes with Ascomycete KOG annotations were annotated as *Phialocephala*, in contrast to roots on *L*, where only 18 of 2530 were annotated as *Phialocephala* (Tables 5 and 6). No expression of *Phialocephala-*annotated genes was identified in shoots on *L*, and only 3 *Phialocephala-*annotated genes were shown to be expressed in shoots on *A*, highlighting this organism as a root endophyte characteristic of *A* (Table 6). Accordingly, due to stronger expression on *A*, soil accounts for most of the *Phialocephala* expression variance, further to that a small proportion is explained by plant ecotype (Fig. 7b). Coupled with the greater gene expression of *Phialocephala*-annotated transcripts in roots of *a*, compared to the *l*, when grown on *A*, this presents the hypothesis that *H*. *lanatus* plants native to *A* have plant-microbiome interaction adaptations to *A*. This adaptation involves a close association with an Ascomycete of the genus *Phialocephala*, or closely related to *Phialocephala*, perhaps with various roles in P and N acquisition, cation transport, metal(loid) tolerance and stress and pathogen resistance for this soil.

For roots on *L*, 166 significantly expressed genes were annotated as *Colletotrichum*, 65 of which were upregulated in *l*, compared to *a* (Table 4). This compares with only 63 significantly expressed genes annotated as *Colletotrichum* expressed in roots on *A*, few of which show an ecotype-specific effect on *A* (Table 4). Again, this could point to ecotype-specific plant-microbiome interactions with *Colletotrichum* or related organisms on *L*-adapted *H*. *lanatus* plants, with the interaction perhaps having a beneficial role in aiding Fe acquisition in *L*.

A total of 144 transcripts best annotated as Glomeromycotina with KOG annotations were assembled and expressed (Additional file 3), with 108 of these significantly expressed in roots on *L*, and only 11 in *A* (Additional file 14). Furthermore, there was little difference in root Glomeromycotina expression due to plant ecotype in either soil (Table 4, Additional file 14), indicating greater AM fungal activity in *L*, compared to *A* roots in both ecotypes. As expected, no significant expression of Glomeromycotina-annotated genes was observed in shoots (Table 4, Additional file 14, Fig. 4).

Of the 108 Glomeromycotina KOG-annotated transcripts significantly expressed in *L*, 43 were annotated as involved in cellular process and signalling (posttranslational modification, signal transduction), 22 in information storage and processing (translation), 38 in metabolism (amino acid, carbohydrate, lipid, secondary metabolite, inorganic ion transport, energy production), and 5 annotated as poorly characterized (Additional file 14). These included 3 ferric reductases, which may be involved in Fe acquisition (Additional file 11). Of the 11 Glomeromycotina KOG-annotated transcripts shown to be significantly expressed on *A*, 4 were KOG annotated as cellular processing and signalling, 6 as information storage and processing and 1 as metabolism (Additional file 14). Overall, the number of transcripts identified as expressed by Glomeromycotina was lower than expected, possibly due to there being only one AM fungal genome for annotation of AM fungal transcripts, making it likely that a number of AM fungal expressed transcripts, in particular those AM more distantly related to *R*. *irregularis*, were missed during the iterative annotation procedure.

Protist-annotated transcripts were assembled and KOG annotated, with 1073 of these assigned as Oomycete and 2107 as protists (other) (Additional file 3). Detection of transcripts best annotated as protists (other) and protists (Oomycete) was greater in roots than in shoots, with Oomycetes making up ~ 29% of all root expressed protists on *A*, and ~ 16% on *L*, indicating that Oomycetes were more active in roots on *A* (Fig. 4, Table 4, Additional file 12). More protist-annotated genes were upregulated in roots in *l*, compared to *a*, on both soils (Table 4). This was most pronounced on *A*, with 365 out of 490 root expressed Oomycete-annotated transcripts upregulated in *l* compared to *a*, while in *L* only 12 out of 239 significantly expressed Oomycete-annotated transcripts were upregulated in *l* compared to *a* (Table 4, Additional file 12). A similar trend could be observed for protists (other) annotated transcripts where 443 out of 1183 significantly root expressed transcripts in *A* were upregulated in *l*, compared to *a*, and 80 out of 1254 on *L* (Table 4, Additional file 12). Of the 365 Oomycete-annotated transcripts upregulated in *l* roots compared to *a* roots in *A* soil 119 were KOG annotated with cellular process and signalling, 108 with information storage and processing and 88 with metabolism. Most of these genes were associated with transcription and translation-type processes (49 of these annotated with post-translational modification, 51 with signal transduction, 23 with RNA processing, 17 with transcription, 62 with translation)) (Additional file 12).

Full DESeq2 results and database annotations for microbial-annotated transcripts are shown in Additional file 12 with corresponding sequences in fasta format in Additional file 15.

### Fungal colonization rates assessed using microscopy

**Fig. 8:**
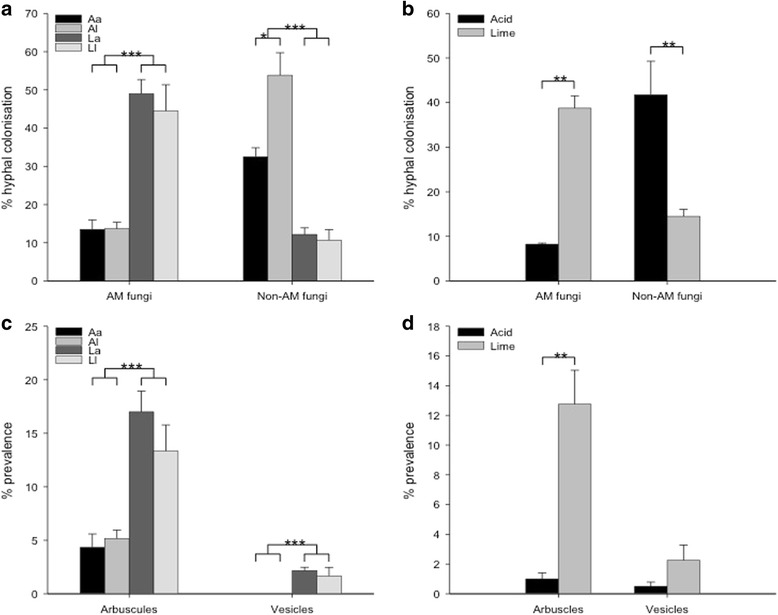
Microscopy-based assessment of % colonization of fungal hyphae and structures in roots of *H*. *lanatus* grown on limestone quarry and acid bog soil. **a** Mean AMF and non-AMF hyphal colonization rates in plants grown in a full factorial reciprocal transplantation design. **b** Mean AMF and non-AMF hyphal colonization rates in plants maintained on their soils of origin, either acid bog or limestone quarry. **c** Mean arbuscule and vesicle prevalence in plants grown in a full factorial reciprocal transplantation design. **d** Mean arbuscule and vesicle prevalence in plants maintained on their soils of origin, either acid bog or limestone quarry. *A* acid bog soil *L* limestone quarry soil, *a* acid bog plant ecotype, *l* limestone quarry plant ecotype; error bars represent standard error. Significant differences are indicated with asterisks, *p <  0.05, **p <  0.01 and ***p < 0.001

Roots of all plants were colonized with both AM and non-AM fungi, whether grown in the reciprocal transplant experiment on *A* or *L* (Fig. 8a, c, Additional file 16) or maintained on their soils of origin (Fig. 8b, d). Hyphal colonization by AM fungi was significantly greater in roots on *L* than *A* in plants maintained on their soil of origin (Fig. 8b) (two-sample *t* test, *t* = − 4.9, df = 5, *p* <  0.01), as well as in plants grown on *L* in the reciprocal transplant experiment (Fig. 8a, Additional file 16) (post hoc Tukey test, *p* < 0.001 following ANOVA, *F*_(1, 21)_ = 65.51, *p* < 0.001). In contrast, hyphal colonization with non-AM fungi was significantly greater in roots from *A* than *L* in plants kept on their soils of origin (Fig. 8b) (two-sample *t* test, *t* = 4.38, df = 6, *p* <  0.01), as well as in plants grown on *A* in the reciprocal transplant experiment (Fig. 8a, Additional file 16) (post hoc Tukey test, *p* <  0.001 following ANOVA, *F*_(1, 20)_ = 72.66, *p* <  0.001). Furthermore, in the reciprocal transplant experiment, *l* showed significantly greater non-AM fungal hyphal colonization compared to *a* when grown on *A* (two-sample *t* test, *t* = − 2.54, df = 8, *p* <  0.05) (Fig. 8a). This is corroborated by a significant interaction effect between soil type and plant ecotype (ANOVA, *F*_(1, 20)_ = 6.06, *p* <  0.05), which indicated that the difference in non-AM hyphal colonization between soil types is greater in *l* than in *a* (Additional file 16). In the transplant experiment, vesicles were only detected in roots grown on *L* (Fig. 8c, Additional file 16). In contrast, arbuscules were detected in roots grown on both soils, with significantly more detected in roots grown on *L* than *A* (ANOVA, *F*_(1, 21)_ = 37.05, *p* <  0.001; post hoc Tukey test, *p* <  0.001) (Fig. 8c, Additional file 16). Images of AM and non-AM fungal structures identified in stained *H*. *lanatus* roots from the reciprocal transplant experiment can be seen in Additional file 17.

## Discussion

In this study, the response of *H*. *lanatus* ecotypes adapted to acid bog or and limestone quarry soil was characterized via meta-transcriptome analysis, complemented by chemical and root staining characterization, to evaluate plant and associated eukaryotic microbiota responses to edaphic stress. The approach is novel, as it assesses natural, multi-species colonization, in distinct genotypes of two disparate *H*. *lanatus* ecotypes, in a reciprocal transplant experiment. This provides insights into edaphic, ecotypic and ecotype-microbiome interaction effects. While RNA-Seq-based analysis of eukaryotic microbiome taxonomies is subject to some limitations, due to the conserved nature of protein coding sequences, and the limited number of fully genome-sequenced fungal and protist species [25], it has the advantage that it can capture information on the active, functional aspects of the microbiome. Incorporation of host and microbiome responses is crucial to understanding plant survival in harsh environments as host-microbe interactions contribute to plant survival, providing symbiont-mediated nutrient acquisition and protection against metal(loid) toxicity and pathogens [75, 76].

The iterative annotation strategy employed, using high-quality protein databases in the absence of a *H*. *lanatus* genome, enabled successful taxonomic and functional assignment of plant and microbe de novo assembled transcripts, based on the closest related organisms present in the database. This enabled us to perform a meta-transcriptome-based gene expression analysis to quantify plant and eukaryotic microbiome responses to extremes of soil, as they differ across the pH range from acid bog to calcareous limestone soil. This approach proved successful in identifying significant soil and ecotype effects, with respect to *H*. *lanatus* root and shoot expressed transcripts. It also provided a means to assess the activity of specific fungal subgroups and Oomycota in *H*. *lanatus* roots and shoots in each soil. Furthermore, it incorporated insights into microbial community composition, but unlike DNA-based amplicon sequencing, it measured fungal and protist microbiome activity in root and shoot rather than presence/absence. Additionally, the approach demonstrated proposed functional redundancy within the microbial community, as demonstrated by the expression of particular genes by multiple genera, such as aldehyde dehydrogenases and ferric reductases.

Plants can utilize a variety of adaptive measures to tolerate the stresses associated with acidic and alkaline soils [1, 34], and the plant gene expression results suggest that *H*. *lanatus* is employing nutrient acquisition and defense strategies in response to *A* and *L*. These observed differences are in line with the differences in nutrient availability and dominant N and P sources for the soils studied. Ammonium is the dominant N source in acid bog soils, and phosphate is fixed by Fe [1]. Calcareous soils are typically poor sources of Fe and P, with phosphate fixed by Ca, and N typically in nitrate form [1], and both ecotypes upregulated genes involved in P, Fe and high-affinity nitrate acquisition on *L* soil. In contrast, P and cation transporters (Cd, Zn, Cu, K, H+) involved in amelioration of abiotic stress responses were upregulated in both ecotypes in *A*. Furthermore, elemental analysis showed that shoot P, As and Mg content was higher in *A* in both ecotypes, with K contents greater in *a* than in *l*, particularly in *A* soil. This corresponded with upregulation of a range of K transporters and homeostasis genes in shoots and roots of *a* on *A* compared to *L* and with upregulation of K channel AKT2 in *a* compared to *l* roots on *A*. AKT2 has been shown to be expressed in root stellar tissue and is reported as key to K^+^ loading and unloading in phloem tissues [77]. This transporter could, therefore, be implicated in the greater K content in *a* compared to *l* shoots on *A*. Maintenance of K homeostasis is well recognized as a stress tolerance mechanism in plants, including acid soil-grown plants, and high-affinity K transporters employed under K starvation have been shown to be regulated by genes responsive to low pH stress and associated toxicities [1, 78]. Increased investment into K homeostasis, and accumulation of K in shoots, therefore, characterizes acid bog soil adapted *H. lanatus*.

Plants can implement various defensive measures to prevent colonization by pathogenic organisms [79]. Initial plant defense involves recognition of fungal elicitors that trigger plant immune responses to prevent colonization. Elicitor signalling involving receptor-like kinases and pattern recognition receptors (PRRs), including PEPR2 [17], that sense either pathogen-associated molecular patterns (PAMPs) or endogenous damage-induced molecular patterns (DAMPs). The observed upregulation of PEPR2 in roots and shoots of *a* on *A* compared to *L* may, therefore, indicate induction of defensive mechanisms in response to pathogen attack in *a* on *A*. Furthermore, lignin biosynthesis is involved in plant response to biotic and abiotic stresses, including nutrient stress and prevention of pathogenic colonization [31, 80]. The stronger expression of lignification genes in both ecotypes on *L* could be in response to limestone soil-specific stressors, such as low P, Fe and N availability as well as biotic factors [80]. In addition to lignification genes, both ecotypes upregulated other pathogen defense-related genes, on *L* compared to *A*. Gene responses affecting the cell wall may have been contributing factors to the observed lower fungal activity in roots on *L* compared to *A* in both ecotypes. Alternatively, the observed lower fungal activity in *L* could simply be due to lower presence of these organisms in this soil. Given the role of root lignification in preventing pathogenic infection [31, 80], upregulation of lignin biosynthesis-related genes in *a* compared to *l* on *A*, could relate to a greater ability of the *a*, than the *l*, to limit soil and pathogen-induced cell damage and infection on *A*, backed up by the use of defensive genes such as PEPR2 in roots and shoots of *a* on *A* compared to *L*. The limestone ecotype, on the other hand, as not adapted to *A*, may be less able to induce this response when grown on this soil type. This hypothesis is based on the observed lower induction of plant genes involved in cell wall lignification, but greater detectable Ascomyctete, Basidiomycete and Oomycete activity in *l* compared to *a* roots in *A*. Most of the Oomycete-annotated transcripts were best annotated as *Phytophthora* and were more active in *A* compared to *L*. *Phytophthora* are major plant pathogens, and cell wall strengthening has previously been proposed as a key method of defense against colonization of these organisms [77], with lignin conferring rigidity to cell walls [80]. Hence, greater expression of lignin biosynthesis-associated genes in *a* may play a role in limiting fungal and Oomycete pathogenic activity in *a* compared to *l* roots, when grown in *A*.

*Phialocephala*-annotated genes were identified as the dominant fungal genera in root gene expression profiles of both ecotypes in *A*, with this genus near absent in roots in *L*. Root endophytes within this genus have previously been reported [81, 82] and can protect against pathogens such as the Oomycete *Phytophthora* [82] which was more active in *A* than in *L*. Within roots grown on *A*, many *Phialocephala*-annotated transcripts were upregulated in *a* compared to *l*, while the opposite effect was observed for Oomycetes and Basidiomycetes, and some other Ascomycete genera. Whether this is mediated via *Phialocephala*-induced cell wall strengthening to prevent pathogen infection, as has been observed in Glomeromycotina-induced pathogen resistance [83], is worth investigation; greater *Phialocephala* expression in *a* compared to *l* on *A* coincided with increased expression of plant lignin-associated genes in *a* compared to *l* on *A*.

*Colletotrichum*-annotated transcripts were more prevalent in *L*-grown roots compared to *A*-grown roots. Within roots from *L*, many *Colletotrichum* and some other Ascomycete genera (including *Fusarium*, *Acremonium* and *Trichoderma*) annotated transcripts were upregulated in *l* compared to *a*. The genus *Colletotrichum* contains mostly pathogenic, but some mutualistic endophytes [84], with symbiotic interactions shown to involve improved P nutrition [85] or production of anti-fungal compounds [86]. It is worth investigating the role of these fungi in plant nutrition and defense in *H*. *lanatus* roots on limestone quarry soil and whether they contribute to plant nutrition in this low P availability environment, particularly since some *Fusarium* species have been identified as endophytes in some plants, including *H*. *lanatus* [27, 87].

Just as plants respond to their edaphic environment, so do fungi, initiating nutrient uptake and other stress response measures [75]. Calcareous soils are typically limited in bioavailable Fe [88], so the greater prevalence of Ascomycota ferric reductases in roots from *L*, could be an adaptation to stresses associated with this soil type. Whether the non-AM fungi then provide Fe to the plant would be worth further investigation. Both non-AM and AM displayed clear soil type effects on colonization and gene expression activity, with greater numbers of significantly expressed Glomeromycotina annotated transcripts and AM hyphae identified in roots grown in *L* compared to *A* soil. *H*. *lanatus* colonization percentages of 25–50% found here by staining in the *L* are consistent with other studies [39]. Lower levels of AM colonization in roots from *A* (~ 10%), plus lower levels of Glomeromycotina activity identified by RNA-Seq are typical, as AM fungi are less prevalent in highly acidic soil such as the acid bog soil *A* used here [5, 89], with some studies indicating sometimes no presence of AM fungi in highly acidic soil [89]. AM fungi have been shown to occur in a wide variety of soil types ranging from pasture to acid peat, with community composition most strongly influenced by pH, rain and soil type [90]. Both *H*. *lanatus* ecotypes responded to low P availability on the *L* by upregulating genes involved in P uptake, transport and increased P use efficiency, suggesting this as an adaptation to *L* in both ecotypes, with low P also known to stimulate colonization and symbiotic action by AM [91]. With further additions of Glomeromycotina genomes to publicly available databases, metatranscriptome analysis as presented here, will most likely become more effective with respect to the identification of functional responses in AM fungi. That Glomeromycotina were contributing to P acquisition in both ecotypes in *L* is displayed by upregulation of genes involved in forming and maintaining the symbiosis, plus genes directly involved in P acquisition from the interaction. Furthermore, the gene expression profile of Glomeromycotina-annotated transcripts in *L* suggests a potential role for AM fungi in Fe nutrition, with expression of AM ferric reductases in *L*. Improved plant Fe nutrition, mediated via AM fungi and involving ferric reductases, has previously been reported, with Fe nutrition of plants particularly positively affected by AM fungi under high pH conditions, and in more sandy soils [92], and this could, therefore, be an important function of AM fungi in *L*, in addition to improved P nutrition. In our study, the number of transcripts observed for Glomeromycotina was comparably low, potentially because there is only one publicly available sequenced genome for Glomeromycotina [57] and, therefore, transcripts from a range of Glomeromycotina may be missed by the BLASTx-based annotation, and hence not included in the overall meta-transcriptome.

Non-AM fungal colonization has been shown to increase tolerance to enhanced bioavailability of toxic metal(loid)s under acidic soil conditions [11], and here, *Phialocephala* HMT1 and a considerable number of genes involved in repair and stress tolerance were upregulated in *A*. Non-AM fungi, including *Phialocephala*, can also provide nutrition benefits to the host in stressful soils, highly acidic soils and in conditions where N is predominantly locked up in organic forms [6], via breakdown into available N sources, i.e. priming [2, 6, 81]. This could be relevant the role of such fungi in plants grown on the acid bog soil, particularly since a number of *Phialocephala* amino acid transport-associated genes were strongly expressed in *A*, and within this soil more strongly upregulated in *a* than *l*. In this study, *Phialocephala*-annotated transcripts upregulated in roots in *A* included arginase, asparagine and d-aspartate oxidase, with arginase, furthermore, identified as being upregulated in *a* compared to *l* in *A*. This is relevant in this context as arginase, asparagine and d-aspartate oxidase have all been shown to play a role in release of N from organic sources [93–95], and it can be proposed that *Phialocephala* may, therefore, provide the plant with access to N from organic sources in *A*. The upregulation of *Phialocephala* K homeostasis genes in roots from *A* compared to *L*, combined with the upregulation of *Phialocephala* K^+^/H^+^ antiporter in *a* compared to *l* on *A*, suggests that soil-specific fungi are using K homeostasis to combat edaphic stress, as was proposed in plants. It is, therefore, proposed that non-AM fungal colonization may benefit *H*. *lanatus*, with this study pointing in particular to a possible beneficial role of organisms closely related to *Phialocephala* in *A*. Although a range of species of *Phialocephala* are documented as beneficial to plants, the genomes of some species have also been shown to contain elements common to pathogenic or saprotrophic lifestyles [96], with some *Phialocephala* shown to cause disease in grasses [97]. Furthermore, the nature of plant-fungal interactions are complex [13, 98] and can be influenced by soil type, plant ecotype and time, with neutral and symbiotic endophytes potentially turning into saprophytes once seasonally mediated plant senescence sets in [75]. The proposed beneficial endophytic role of *Phialocephala* in our phenotypically healthy-looking *H*. *lanatus* plants in acid bog soil is therefore subject to further investigation, and pathogenic activity of these organisms at some point during the lifecycle of the plants cannot be ruled out.

Fungal symbionts are implicated in enhancing plant abiotic stress tolerance and facilitate stress tolerance in plants via habitat-adapted symbiosis [99], with significant three-way interaction effect on cumulative shoot weight (endophyte × ecotype × soil) previously reported in *Festuca arundinacea* [100]. In our full factorial, reciprocal soil transplant investigation, we have shown that, albeit to a lesser degree than soil type, host ecotype can also influence non-AM fungi colonization and activity.

## Conclusions

Fungal and Oomycete activity was higher in roots grown in our organic-rich acid bog soil compared to the minerogenic limestone soil, and low levels of fungal and Oomycete activity were observed in all shoots. Ascomycota showed the highest level of activity in roots grown in both soils, but there were strong soil and ecotype-specific differences with respect to the activity of different Ascomycete genera. *Phialocephala*-annotated transcripts dominated in roots in acid bog soil, and many of these transcripts were upregulated in roots in the acid ecotype compared to limestone ecotype plants. In contrast, a very mixed group of Ascomycete genera were shown to be active in limestone soil-grown roots, with the highest level observed for *Colletotrichum*-annotated transcripts. Furthermore, *Colletotrichum*, a range of other Ascomycota genera, Basidiomycetes and Oomycetes showed higher levels of activity in limestone ecotype roots compared to acid ecotype roots on both soils. Lignin biosynthesis genes were upregulated on limestone soil, and on acid bog soil they were upregulated in acid ecotype compared to limestone ecotype plants. Our results imply a possible role of lignin biosynthesis in limiting fungal and Oomycete activity in roots in the acid ecotype compared to limestone ecotype plants, when grown in acid bog soil, perhaps induced by the root endophyte *Phialocephala*. As expected, AM-fungi were shown to be more active in the pH 7.5 mineral limestone soil compared to the pH 3.5 organic acid bog soil, and this was the case in roots of both plant ecotypes. The same applied to a range of other Ascomycete genera, including *Colletotrichum*. The transcriptomics data suggested that both AM and non-AM fungi of some Ascomycota genera may play a role in P and Fe nutrition in the limestone soil, while other non-AM Ascomycota, in particular *Phialocephala-*related organisms, may aid plant N and K nutrition and increase tolerance to metal(loid) ions in the acid bog soil. In *H*. *lanatus* shoots, fungal transcripts were predominantly Ascomycete annotated and showed low levels of activity, with numbers slightly higher in acid bog compared to limestone quarry soil-grown shoots, corroborating the observation that there was no systemic fungal disease in the plants at the time of harvest. Our meta-transcriptome analyses provided insights into the functional and taxonomic eukaryotic microbiota community composition and interaction within two contrasting *H*. *lanatus* ecotypes. With natural multi-species eukaryotic plant microbiomes so far poorly characterized, our results in this particularly stress resistant and phenotypically plastic plant species outline a novel approach towards a more holistic study of edaphic stress adaptation.

## Additional files

Additional file 1: Description of additional RNA-Seq data obtained from 3 hydroponically grown plants that were integrated into the *Holcus lanatus* metatranscriptome assembly. (DOCX 17 kb)

Additional file 2: Elemental composition, pH and loss on ignition (LoI) of acid bog and limestone quarry soils. (DOCX 20 kb)

Additional file 3: The meta-transcriptome assembly: The number of assembled plant and microbial transcripts with KOG annotations. (DOCX 21 kb)

Additional file 4: Significant DAVID GO enrichment results obtained for the 16 up-regulated and down-regulated plant gene lists. (XLSX 121 kb)

Additional file 5: Extended REViGO results for the 16 up-regulated and down-regulated plant gene lists. (XLSX 68 kb)

Additional file 6: Selected plant expressed gene results, for those genes discussed in the text. The table contains the following information for selected plant-assigned transcripts that are discussed in the text: 1) Plant root/shoot (for reported DESeq2 pairwise comparison); 2) Transcript ID; 3) gene description; 4) function; 5) possible role; 6) relevant reference (citations for relevant references are contained in Additional file 7); 7) DESeq2 result: Al-v-Ll = Acid bog soil limestone ecotype versus Limestone soil limestone ecotype, Aa-v-Al = Acid bog soil acid ecotype versus Acid bog soil limestone ecotype, La-v-Ll = Limestone soil acid ecotype versus Limestone soil limestone ecotype; 8) Two columns to indicate whether the transcript was significantly upregulated in acid or limestone soil and/or acid or limestone ecotype. (XLSX 23 kb)

Additional file 7: Additional references cited in Additional file 6 and Additional file 11. (DOCX 32 kb)

Additional file 8: Plant-assigned transcripts, full results table, annotation and DESeq2 analysis. The table contains the following information for each expressed plant-assigned transcript: 1) The annotation of transcripts: this includes functional annotation (KOG class, KOG group, KOG definition) and best BLAST match against plant-refseq (match accession number, match description, percentage ID, BLAST score, length of alignment and percentage sequence coverage); 2) The full DESeq2 result for each pairwise comparison in shoot (list 1-4) and root (list 5-8): these columns contain the log_2_foldchange and adjusted *P* value (FDR) for pairwise comparisons. Pairwise comparisons are coded as follows: soil (*A* = acid bog soil, *L* = limestone quarry soil), plant ecotype (*a* = acid bog plant ecotype, *l* = limestone quarry plant ecotype) in shoots and roots: Shoot Aa-v-La = Acid bog soil acid ecotype versus Limestone soil acid ecotype, Shoot Al-v-Ll = Acid bog soil limestone ecotype versus Limestone soil limestone ecotype, Shoot Aa-v-Al = Acid bog soil acid ecotype versus Acid bog soil limestone ecotype, Shoot La-v-Ll = Limestone soil acid ecotype versus Limestone soil limestone ecotype, Root Aa-v-La = Acid bog soil acid ecotype versus Limestone soil acid ecotype, Root Al-v-Ll = Acid bog soil limestone ecotype versus Limestone soil limestone ecotype, Root Aa-v-Al = Acid bog soil acid ecotype versus Acid bog soil limestone ecotype, Root La-v-Ll = Limestone soil acid ecotype versus Limestone soil limestone ecotype; 3) The count table; 4) Additional BLAST annotations showing the best BLAST match against *Arabidopsis lyrata, Arabidopsis thaliana* and *Brachypodium distachyon* databases (sequence length, match accession number, e-value, percentage ID, BLAST score and length of alignment). (XLSX 18037 kb)

Additional file 9: Plant transcripts in fasta format. Transcript-ids in the fasta file correspond to those described in Additional file 8. Compressed .tar.gz text file. (GZ 8552 kb)

Additional file 10: Additional qPCR information. Targets and primers used for qPCR and comparison of results obtained with RNA-Seq and qPCR. (DOCX 22 kb)

Additional file 11: Selected fungal annotated transcript results, including those discussed in the text. The table contains the following information for selected microbial assigned transcripts that are expressed in *Holcus lanatus* roots, many of which discussed in the text: 1) Transcript ID; 2) Best BLAST match (taxonomic division, genus and gene description); 3) KOG annotations (KOG ID, KOG group, KOG class, KOG definition); 4) possible role; 5) relevant reference (citations for relevant references are contained in Additional file 7); 6) two columns to indicate whether the transcript was significantly upregulated in acid or limestone soil, acid or limestone ecotype; 7) The full DESeq2 result for each root pairwise comparison: these columns contain the log_2_foldchange and adjusted P value (FDR) for root pairwise comparisons. Pairwise comparisons are coded as follows: soil (*A* = acid bog soil, *L* = limestone quarry soil), plant ecotype (*a* = acid bog plant ecotype, *l* = limestone quarry plant ecotype): Root Aa-v-La = Acid bog soil acid ecotype versus Limestone soil acid ecotype, Root Al-v-Ll = Acid bog soil limestone ecotype versus Limestone soil limestone ecotype, Root Aa-v-Al = Acid bog soil acid ecotype versus Acid bog soil limestone ecotype, Root La-v-Ll = Limestone soil acid ecotype versus Limestone soil limestone ecotype. (XLSX 35 kb)

Additional file 12: Microbial-assigned transcripts, full results table, annotation and DESeq2 analysis. Description of File: The table includes information on expressed microbial transcripts. 1) Phylogenetic annotation of each transcript; 2) Whether significantly expressed in root acid bog soil (RA), root limestone soil (RL), shoot acid bog soil (SA) and/or shoot limestone soil (SL). Significantly expressed transcripts is defined as the number of microbial annotated transcripts that obtained ≥5 aligned reads in at least 3 samples in each of the treatments RA (total 8 samples), RL (total 9 samples), SA (total 10 samples), SL (total 9 samples); 3) two columns indicating whether the transcript is “upregulated in acid or limestone ecotype in acid bog soil” and/or “upregulated in acid or limestone ecotype in limestone soil” 4) KOG functional annotation (KOG ID, KOG definition, KOG class, KOG group) and best BLAST match (sequence length, database that contained the best match, accession number, gene description, best match organism, e-value, percentage ID, BLAST score, percentage sequence coverage); 5) The full DESeq2 result for each pairwise comparison in shoot (list 1-4) and root (list 5-8): these columns contain the log_2_foldchange and adjusted P value (FDR) for pairwise comparisons. Pairwise comparisons are coded as follows: soil (*A* = acid bog soil, *L* = limestone quarry soil), plant ecotype (*a* = acid bog plant ecotype, *l* = limestone quarry plant ecotype), shoots and roots (Shoot, Root), see sheet “DESeq2 pairwise comparisons” for further explanation of the 8 pairwise comparisons; 6) The count table. (XLSX 6498 kb)

Additional file 13: Significantly expressed and KOG annotated Basidiomycete transcripts in roots and shoots in each soil. The number of significantly expressed and KOG annotated Basidiomycete transcripts (All) is defined as the number of transcripts that obtained ≥5 aligned reads in at least 3 samples from each of the following treatments: root acid bog soil (RA), root lime soil (RL), shoot acid bog soil (SA) and shoot lime soil (SL). Significant ecotype effects (absolute log_2_FC ≥ 1 or ≤ − 1, FDR < 0.05) as identified by DESeq2 analysis are reported in subsequent columns for each treatment group, under the heading plant effect. (DOCX 27 kb)

Additional file 14: Significantly expressed and KOG annotated Glomeromycotina transcripts in root and shoot in each soil. The number of significantly expressed and KOG annotated Glomeromycotina transcripts (All) is defined as the number of transcripts that obtained ≥5 aligned reads in at least 3 samples from each of the following treatments: root acid bog soil (RA), root lime soil (RL), shoot acid bog soil (SA) and shoot lime soil (SL). Significant ecotype effects (absolute log_2_FC ≥ 1 or ≤ − 1, FDR <  0.05) as identified by DESeq2 analysis are reported in subsequent columns for each treatment group, under the heading plant effect. (DOCX 27 kb)

Additional file 15: Microbial transcripts in fasta format. Transcript-ids in the fasta file correspond to those described in Additional file 12. Compressed .tar.gz text file. (GZ 2398 kb)

Additional file 16: Colonization percentages of AM hyphae, non-AM hyphae, arbuscules and vesicles, in acid bog and limestone quarry ecotypes of *H. lanatus*, grown in a reciprocal soil transplantation design. (*A* = acid bog soil, *L* = limestone quarry soil), plant ecotype (*a* = acid bog plant ecotype, *l* = limestone quarry plant ecotype). (DOCX 18 kb)

Additional file 17: AM and non-AM fungal structures of stained *H. lanatus* roots of (a, b) acid bog ecotype on acid bog soil; (c, d) limestone quarry ecotype on acid bog soil; (e, f) acid bog ecotype on limestone quarry soil; (g, h) limestone quarry ecotype on limestone quarry soil. (DOCX 93871 kb)

## Abbreviations

*A*: Acid bog soil
*Aa*: Acid ecotype grown on acid bog soil
*Al*: Limestone ecotype on acid bog soil
AM: Arbuscular mycorrhiza/l
ANOVA: Analysis of variance
Arf1: GTP-binding ADP-ribosylation factor
BLASTx: Basic local alignment search tool
bp: Base pairs
cDNA: Complementary deoxyribonucleic acid
DAMPS: Damage-induced molecular patterns
DEGs: Differentially expressed genes
DNA: Deoxyribonucleic acid
FDR: False discovery rate
GLM: General linear models
GO: Gene Ontology
ICP-MS: Inductively coupled plasma mass spectrometry
JA: Jasmonic acid
JGI: The Genome Portal of the Department of Energy Joint Genome Institute
KOG: EuKaryotic Orthologous Groups
KUP1: Potassium transporter 1
*L*: Lime stone quarry soil
*La*: Acid ecotype on limestone soil
*Ll*: Limestone ecotype on limestone soil
log_2_FC: Log_2_fold change
LUX: Luminous flux per unit area
NCBI: National Center for Biotechnology Information
NGS: Next-generation sequencing
Nr: Non-redundant
PAMPs: Pathogen-associated molecular patterns
PEPR2: Plasma membrane leucine-rich repeat receptor kinase 2
pmPOX2b: Plasma membrane-bound peroxidase 2b
PRRs: Pattern recognition receptors
PT: Phosphate transporter
qPCR: Quantitative polymerase chain reaction
RA: Root acid bog soil
RefSeq: Reference sequence
RL: Root limestone soil
RNA: Ribonucleic acid
RNA-Seq: Ribonucleic acid sequencing
Rpm: Revolutions per minute
RT: Reverse transcription
SA: Shoot acid bog soil
SAc: Salicylic acid
SL: Shoot limestone soil
