# Supplementary material for: Microbiome and ecotypic adaption of Holcus lanatus (L.) to extremes of its soil pH range, investigated through transcriptome sequencing
Source: Microbiome. 2018 Mar 20;6:48. doi: 10.1186/s40168-018-0434-3 (PMC5859661; doi:10.1186/s40168-018-0434-3)
Supplement: Supplementary file 1 — Description of additional RNA-Seq data obtained from 3 hydroponically grown plants that were integrated into the Holcus lanatus metatranscriptome assembly. (DOCX 17 kb) [file 40168_2018_434_MOESM1_ESM.docx]

**Additional file 1:** Description of additional RNA-Seq data obtained from 3 hydroponically grown plants that were integrated into the *Holcus lanatus* metatranscriptome assembly.

Intact *H. lanatus*, shoot and root ball, plants were collected along with topsoil from 3 locations in Northern Ireland; a disused limestone quarry pH 7.5, Map. Ref. NR 23472 02816 “limestone soil”; an acidic peat bog pH 3.5, Map. Ref. NW 02918 19660 “acid bog”; a rough grassland pH 5.5, Map. Ref. NW 60194 33732 “intermediate”. Hydroponically grown plants, only used for inclusion in the meta-transcriptome assembly, were cultivated as follows: A rootless tiller of 1 individual from each of the acid, limestone and intermediate ecotypes was transferred to 0.5mM CaCl_2_ solution. Plants were equilibrated for 10 days in a growth chamber set at 25°C, 12 hour day:night cycle and 5000 LUX (Panasonic, Japan). Tillers were then transferred for 48 hours to either 50μM AlCl_3_ solution (acid and limestone ecotypes) or fresh 0.5mM CaCl_2_ solution (intermediate ecotype) before harvesting, using the same procedure as soil-grown plants. Root samples were ground to a fine powder using 1.5ml microcentrifuge pestles and ≤ 106μm acid washed glass beads under liquid nitrogen. RNA was extracted using a variation of the phenol-chloroform procedure [Hot phenol RNA extraction protocol, Mylne, 2012, <http://www.mylne.org/files/Mini_Hot_Phenol.pdf>], followed by DNase treatment using TURBO DNA-free kit (ThermoFisher Scientific, USA). RNA quality controlled was ascertained using a Nanodrop 8000 spectrophotometer (ThermoFisher Scientific) and an Agilent 2200 Tape Station (Agilent Technologies, USA). Barcoded 125bp paired-end libraries (polyA selected, to enrich for eukaryotic mRNA and remove rRNA, Illumina TrueSeq) were generated and sequenced at the Earlham Institute (UK) on an Illumina HiSeq 2500. Samples were sequenced at 10 per lane (together with the RNAseq of the transplant experiment see main text). The RNA-Seq data of the roots of the 3 hydroponically grown plants (fastq files) is publicly available in ArrayExpress under accession E-MTAB-4014 at <https://www.ebi.ac.uk/arrayexpress/E-MTAB-4014>. RNA-Seq data from the hydroponically grown plants was only used, together with the other RNA-Seq data, for the generation of the meta-transcriptome, but did not form part of the gene expression analysis, hence is not reported on in the results section of the main paper.
