# Supplementary material for: Microbiome and ecotypic adaption of Holcus lanatus (L.) to extremes of its soil pH range, investigated through transcriptome sequencing
Source: Microbiome. 2018 Mar 20;6:48. doi: 10.1186/s40168-018-0434-3 (PMC5859661; doi:10.1186/s40168-018-0434-3)
Supplement: Supplementary file 2 — Elemental composition, pH and loss on ignition (LoI) of acid bog and limestone quarry soils. (DOCX 20 kb) [file 40168_2018_434_MOESM2_ESM.docx]

**Additional file 2:** Elemental composition, pH and loss on ignition (LoI) of acid bog and limestone quarry soils.

|  |  | **Mean content (mg/kg)** | | **Standard error** | |
| --- | --- | --- | --- | --- | --- |
| **Element** | ***p*-value** | **Acid Bog** | **Limestone** | **Acid Bog** | **Limestone** |
| Al^a^ | < 0.001 | 698 | 43402 | 11.3 | 135 |
| As^b^ | < 0.001 | 1.36 | 7.5 | 0.01 | 0.05 |
| Ba^b^ | < 0.001 | 5.75 | 198.78 | 0.07 | 3.17 |
| Bi^a^ | < 0.01 | 0.04 | 0.1 | 0 | 0 |
| Ca^c^ | < 0.01 | 5564 | 59265 | 198 | 1287 |
| Cd^b^ | < 0.001 | 0.07 | 0.24 | 0 | 0 |
| Co^a^ | < 0.001 | 0.32 | 11.96 | 0 | 0.11 |
| Cr^a^ | < 0.01 | 2.07 | 85.84 | 0.07 | 2.39 |
| Cs^b^ | < 0.001 | 0.04 | 3.55 | 0 | 0.02 |
| Cu^c^ | < 0.01 | 4.28 | 23.47 | 0.37 | 0.92 |
| Li^b^ | < 0.001 | 0.63 | 43.42 | 0.01 | 0.12 |
| Mg^b^ | < 0.001 | 3122.2 | 9268.7 | 9.6 | 47.8 |
| Mn^b^ | < 0.001 | 20.21 | 838.66 | 0.03 | 5.41 |
| Mo^a^ | < 0.05 | 0.11 | 0.34 | 0.01 | 0.03 |
| Ni^a^ | < 0.001 | 1.53 | 35.36 | 0.04 | 0.21 |
| P^a^ | < 0.001 | 156.07 | 624.03 | 2.28 | 3.61 |
| Pb^a^ | < 0.001 | 4.16 | 19.53 | 0.03 | 0.25 |
| Rb^c^ | < 0.001 | 2.36 | 79.77 | 0.26 | 0.29 |
| S^c^ | < 0.01 | 2250 | 532.33 | 92.9 | 4.06 |
| Sr^b^ | < 0.001 | 29.23 | 157.58 | 0.25 | 1.29 |
| Ti^c^ | < 0.001 | 21.1 | 5367 | 1.61 | 50 |
| U^b^ | < 0.001 | 0.03 | 0.79 | 0 | 0.01 |
| V^a^ | < 0.001 | 1.84 | 102.3 | 0.05 | 0.41 |
| Zn^c^ | < 0.001 | 15.8 | 78.7 | 0.46 | 1.24 |
| pH | < 0.001 | 3.71 | 7.71 | 0.01 | 0.002 |
| LoI | < 0.001 | 97.2% | 5.8% | 7.88% | 3.47% |

Extraction and detection method indicated by ^a^ ICP-MS with nitric acid + hydrochloric acid digestion, ^b^ ICP-MS with nitric acid digestion or ^c^ X-Ray fluorescence
