# Supplementary material for: Microbiome and ecotypic adaption of Holcus lanatus (L.) to extremes of its soil pH range, investigated through transcriptome sequencing
Source: Microbiome. 2018 Mar 20;6:48. doi: 10.1186/s40168-018-0434-3 (PMC5859661; doi:10.1186/s40168-018-0434-3)
Supplement: Supplementary file 3 — The meta-transcriptome assembly: The number of assembled plant and microbial transcripts with KOG annotations. (DOCX 21 kb) [file 40168_2018_434_MOESM3_ESM.docx]

**Additional file 3:** The meta-transcriptome assembly: The number of assembled plant and microbial transcripts with KOG annotations

| \|  \| Plant \| Protozoa \| \| Fungi \| \| \| \| Ascomycetes \| \| \| \| \| \| \| --- \| --- \| --- \| --- \| --- \| --- \| --- \| --- \| --- \| --- \| --- \| --- \| --- \| --- \| \| KOG annotated transcripts expressed at >=5 reads in >=3 out of 36 samples \| *Holcus lanatus* \| Protozoa (Oomycetes) \| Protozoa (Other) \| Ascomycetes \| Basidiomycota \| Glomeromycotina \| Fungi (Other) \| *Phialocephala* \| *Colletotrichum* \| *Fusarium* \| *Acremonium* \| *Trichoderma* \| Ascomycetes Other \| \| CELLULAR PROCESSES AND SIGNALING \| 5528 \| 440 \| 779 \| 1599 \| 139 \| 50 \| 77 \| 373 \| 64 \| 60 \| 24 \| 30 \| 1048 \| \| Cell motility \| 1 \| 2 \| 1 \| 1 \| 0 \| 0 \| 0 \| 1 \| 0 \| 0 \| 0 \| 0 \| 0 \| \| Cell wall/membrane/envelope biogenesis \| 209 \| 14 \| 10 \| 110 \| 7 \| 2 \| 0 \| 23 \| 4 \| 7 \| 1 \| 0 \| 75 \| \| Cytoskeleton \| 446 \| 67 \| 189 \| 155 \| 27 \| 9 \| 15 \| 37 \| 9 \| 2 \| 0 \| 2 \| 105 \| \| Defense mechanisms \| 211 \| 5 \| 0 \| 30 \| 0 \| 1 \| 0 \| 10 \| 0 \| 2 \| 2 \| 0 \| 16 \| \| Extracellular structures \| 13 \| 0 \| 1 \| 8 \| 0 \| 0 \| 0 \| 5 \| 0 \| 0 \| 0 \| 0 \| 3 \| \| Intracellular trafficking, secretion, and vesicular transport \| 604 \| 66 \| 77 \| 222 \| 11 \| 5 \| 10 \| 75 \| 8 \| 8 \| 4 \| 1 \| 126 \| \| Nuclear structure \| 115 \| 5 \| 1 \| 19 \| 0 \| 0 \| 0 \| 6 \| 0 \| 0 \| 1 \| 0 \| 12 \| \| Posttranslational modification, protein turnover, chaperones \| 1708 \| 163 \| 391 \| 676 \| 69 \| 21 \| 41 \| 115 \| 25 \| 27 \| 10 \| 15 \| 484 \| \| Signal transduction mechanisms \| 2221 \| 118 \| 109 \| 378 \| 25 \| 12 \| 11 \| 101 \| 18 \| 14 \| 6 \| 12 \| 227 \| \| INFORMATION STORAGE AND PROCESSING \| 3210 \| 274 \| 744 \| 1475 \| 133 \| 34 \| 125 \| 324 \| 53 \| 87 \| 26 \| 27 \| 958 \| \| Chromatin structure and dynamics \| 182 \| 11 \| 28 \| 68 \| 3 \| 1 \| 4 \| 18 \| 6 \| 2 \| 1 \| 3 \| 38 \| \| Replication, recombination and repair \| 352 \| 17 \| 6 \| 54 \| 1 \| 4 \| 2 \| 43 \| 1 \| 0 \| 2 \| 0 \| 8 \| \| RNA processing and modification \| 575 \| 56 \| 67 \| 185 \| 9 \| 7 \| 8 \| 86 \| 5 \| 9 \| 0 \| 1 \| 84 \| \| Transcription \| 936 \| 58 \| 17 \| 166 \| 5 \| 0 \| 2 \| 64 \| 2 \| 9 \| 2 \| 1 \| 88 \| \| Translation, ribosomal structure and biogenesis \| 1165 \| 132 \| 626 \| 1002 \| 115 \| 22 \| 109 \| 113 \| 39 \| 67 \| 21 \| 22 \| 740 \| \| METABOLISM \| 4600 \| 260 \| 452 \| 2100 \| 117 \| 48 \| 56 \| 604 \| 85 \| 111 \| 63 \| 51 \| 1186 \| \| Amino acid transport and metabolism \| 631 \| 55 \| 58 \| 316 \| 18 \| 8 \| 8 \| 117 \| 8 \| 16 \| 9 \| 10 \| 156 \| \| Carbohydrate transport and metabolism \| 796 \| 46 \| 53 \| 371 \| 15 \| 6 \| 12 \| 91 \| 20 \| 21 \| 5 \| 9 \| 225 \| \| Cell cycle control, cell division, chromosome partitioning \| 321 \| 12 \| 27 \| 118 \| 5 \| 0 \| 2 \| 46 \| 4 \| 1 \| 0 \| 6 \| 61 \| \| Coenzyme transport and metabolism \| 163 \| 14 \| 44 \| 68 \| 7 \| 5 \| 2 \| 25 \| 3 \| 2 \| 1 \| 1 \| 36 \| \| Energy production and conversion \| 780 \| 71 \| 191 \| 608 \| 51 \| 11 \| 23 \| 94 \| 24 \| 51 \| 19 \| 15 \| 405 \| \| Inorganic ion transport and metabolism \| 402 \| 24 \| 19 \| 160 \| 10 \| 5 \| 2 \| 38 \| 8 \| 8 \| 5 \| 3 \| 98 \| \| Lipid transport and metabolism \| 624 \| 13 \| 30 \| 225 \| 8 \| 8 \| 1 \| 86 \| 9 \| 5 \| 16 \| 4 \| 105 \| \| Nucleotide transport and metabolism \| 167 \| 12 \| 26 \| 66 \| 3 \| 1 \| 3 \| 33 \| 1 \| 2 \| 0 \| 0 \| 30 \| \| Secondary metabolites biosynthesis, transport and catabolism \| 716 \| 13 \| 4 \| 168 \| 0 \| 4 \| 3 \| 74 \| 8 \| 5 \| 8 \| 3 \| 70 \| \| POORLY CHARACTERIZED \| 3401 \| 99 \| 132 \| 819 \| 16 \| 12 \| 13 \| 319 \| 33 \| 25 \| 20 \| 8 \| 414 \| \| Function unknown \| 769 \| 26 \| 14 \| 115 \| 2 \| 3 \| 0 \| 78 \| 2 \| 0 \| 3 \| 0 \| 32 \| \| General function prediction only \| 2632 \| 73 \| 118 \| 704 \| 14 \| 9 \| 13 \| 241 \| 31 \| 25 \| 17 \| 8 \| 382 \| \| TOTAL KOG ANNOTATED \| 16739 \| 1073 \| 2107 \| 5993 \| 405 \| 144 \| 271 \| 1620 \| 235 \| 283 \| 133 \| 116 \| 3606 \| |
| --- | --- | --- | --- | --- | --- | --- | --- | --- | --- | --- | --- | --- | --- | --- | --- | --- | --- | --- | --- | --- | --- | --- | --- | --- | --- | --- | --- | --- | --- | --- | --- | --- | --- | --- | --- | --- | --- | --- | --- | --- | --- | --- | --- | --- | --- | --- | --- | --- | --- | --- | --- | --- | --- | --- | --- | --- | --- | --- | --- | --- | --- | --- | --- | --- | --- | --- | --- | --- | --- | --- | --- | --- | --- | --- | --- | --- | --- | --- | --- | --- | --- | --- | --- | --- | --- | --- | --- | --- | --- | --- | --- | --- | --- | --- | --- | --- | --- | --- | --- | --- | --- | --- | --- | --- | --- | --- | --- | --- | --- | --- | --- | --- | --- | --- | --- | --- | --- | --- | --- | --- | --- | --- | --- | --- | --- | --- | --- | --- | --- | --- | --- | --- | --- | --- | --- | --- | --- | --- | --- | --- | --- | --- | --- | --- | --- | --- | --- | --- | --- | --- | --- | --- | --- | --- | --- | --- | --- | --- | --- | --- | --- | --- | --- | --- | --- | --- | --- | --- | --- | --- | --- | --- | --- | --- | --- | --- | --- | --- | --- | --- | --- | --- | --- | --- | --- | --- | --- | --- | --- | --- | --- | --- | --- | --- | --- | --- | --- | --- | --- | --- | --- | --- | --- | --- | --- | --- | --- | --- | --- | --- | --- | --- | --- | --- | --- | --- | --- | --- | --- | --- | --- | --- | --- | --- | --- | --- | --- | --- | --- | --- | --- | --- | --- | --- | --- | --- | --- | --- | --- | --- | --- | --- | --- | --- | --- | --- | --- | --- | --- | --- | --- | --- | --- | --- | --- | --- | --- | --- | --- | --- | --- | --- | --- | --- | --- | --- | --- | --- | --- | --- | --- | --- | --- | --- | --- | --- | --- | --- | --- | --- | --- | --- | --- | --- | --- | --- | --- | --- | --- | --- | --- | --- | --- | --- | --- | --- | --- | --- | --- | --- | --- | --- | --- | --- | --- | --- | --- | --- | --- | --- | --- | --- | --- | --- | --- | --- | --- | --- | --- | --- | --- | --- | --- | --- | --- | --- | --- | --- | --- | --- | --- | --- | --- | --- | --- | --- | --- | --- | --- | --- | --- | --- | --- | --- | --- | --- | --- | --- | --- | --- | --- | --- | --- | --- | --- | --- | --- | --- | --- | --- | --- | --- | --- | --- | --- | --- | --- | --- | --- | --- | --- | --- | --- | --- | --- | --- | --- | --- | --- | --- | --- | --- | --- | --- | --- | --- | --- | --- | --- | --- | --- | --- | --- | --- | --- | --- | --- | --- | --- | --- | --- | --- | --- | --- | --- | --- | --- | --- | --- | --- | --- | --- | --- | --- | --- | --- | --- | --- | --- | --- | --- | --- | --- | --- | --- | --- | --- | --- | --- | --- | --- | --- | --- | --- | --- | --- | --- | --- | --- | --- | --- | --- | --- | --- | --- | --- | --- | --- |
