# Supplementary material for: Microbiome and ecotypic adaption of Holcus lanatus (L.) to extremes of its soil pH range, investigated through transcriptome sequencing
Source: Microbiome. 2018 Mar 20;6:48. doi: 10.1186/s40168-018-0434-3 (PMC5859661; doi:10.1186/s40168-018-0434-3)
Supplement: Supplementary file 7 — Additional references cited in Additional file 6 and Additional file 11. (DOCX 32 kb) [file 40168_2018_434_MOESM7_ESM.docx]

**Additional file 7:** Additional references cited in Additional file 6 and Additional file 11.

S1: Chanroj S, Lu Y, Padmanaban S, Nanatani K, Uozumi N, Rao R, et al. Plant-specific cation/H+ exchanger 17 and its homologs are endomembrane K+ transporters with roles in protein sorting. J Biol Chem. 2011;286:33931-33941

S2: Kim E, Kwak J, Uozumi N, Schroeder J. AtKUP1: An Arabidopsis gene encoding high-affinity potassium transport activity. Plant Cell. 1998;10:51-62

S3: Fu H, Luan S. AtKUP1: A dual-affinity K+ transporter from Arabidopsis. Plant Cell. 1998;10:63-73

S4: Yang C, Zhao N, Xu C, Liu B, Shi D. Regulation of ion homeostasis in rice subjected to salt and alkali stresses. Aust J Crop Sci. 2012;6:724-731

S5: Wang H, Zhang M, Guo R, Shi D, Liu B, Lin X, et al.. Effects of salt stress on ion balance and nitrogen metabolism of old and young leaves in rice (Oryza sativa L.). BMC Plant Biol. 2012;12:194

S6: Gaymard F, Pilot G, Lacombe B, Bouchez D, Bruneau D, Boucherez J, et al.. Identification and disruption of a plant shaker-like outward channel involved in K+ release into the xylem sap. Cell. 1998;94:647-655

S7: Drechsler N, Zheng Y, Bohner A, Nobmann B, von Wiren N, Kunze R, et al. Nitrate-dependent control of shoot K homeostasis by the Nitrate Transporter1/Peptide Transporter family member NPF7.3/NRT1.5 and the Stelar K+ Outward Rectifier SKOR in Arabidopsis. Plant Physiol. 2015;169:2832-2847

S8: Landmann C, Fink B, Schwab W. FaGT2: a multifunctional enzyme from strawberry (Fragaria x ananassa) fruits involved in the metabolism of natural and xenobiotic compounds. Planta, 2007;226(2):417-428

S9: Seigneurin-Berny D, Gravot A, Auroy P, Mazard C, Kraut A, Finazzi G, et al.. HMA1, a new Cu-ATPase of the chloroplast envelope, is essential for growth under adverse light conditions. J Biol Chem, 2006;281(5):2882-2892

S10: Dixon DP, Lapthorn A, Edwards R. Plant glutathione transferases. Genome Biol. 2002;3:REVIEWS3004

S11: Zhang C, Wu Z, Ju T, Ge Y. Purification and identification of glutathione S-transferase in rice root under cadmium stress. Rice Sci. 2013;20:173-178

S12: Yamaguchi Y, Huffaker A, Bryan AC, Tax FE, Ryan CA. PEPR2 Is a Second Receptor for the Pep1 and Pep2 Peptides and Contributes to Defense Responses in Arabidopsis. Plant Cell, 2010;22, 508-522

S13: Mackey D, Belkhadir Y, Alonso JM, Ecker JR, Dangl JL. Arabidopsis RIN4 is a target of the type III virulence effector AvrRpt2 and modulates RPS2-mediated resistance. Cell. 2003;112:379-389

S14: Xie DX, Feys BF, James S, Nieto-Rostro M, Turner JG. COI1: an Arabidopsis gene required for jasmonate-regulated defense and fertility. Science. 1998;280-1091-1094

S15: Coego A, Ramirez V, Gil MJ, Flors V, Mauch-Mani B, Vera P. An Arabidopsis homeodomain transcription factor, overexpressor of cationic peroxidase 3, mediates resistance to infection by necrotrophic pathogens. Plant Cell. 2005;17;2123-2137

S16: Tani T, Sobajima H, Okada K, Chujo T, Arimura S, Tsutsumi N, et al. Identification of the OsOPR7 gene encoding 12-oxophytodienoate reductase involved in the biosynthesis of jasmonic acid in rice. Planta, 2008;227:517-526

S17: Schaller F, Weiler EW. Enzymes of octadecanoid biosynthesis in plants. FEBS J. 1997; 245:294-299

S18: Zhao Q, Nakashima J, Chen F, Yin Y, Fu C, Yun J, et al.. Laccase is necessary and nonredundant with PEROXIDASE for lignin polymerization during vascular development in Arabidopsis. Plant Cell. 2013a;25:3976-3987

S19: Berthet S, Demont-Caulet N, Pollet B, Bidzinski P, Cézard L, Le Bris P, et al.. Disruption of LACCASE4 and 17 results in tissue-specific alterations to lignification of Arabidopsis thaliana stems. Plant Cell. 2011;23:1124-1137

S20: Wang Y, Bouchabke-Coussa O, Lebris P, Antelme S, Soulhat C, Gineau E, et al. laccase5 is required for lignification of the Brachypodium distachyon culm. Plant Physiol. 2015;168:192-204

S21: Trabucco GM, Matos DA, Lee SJ, Saathoff AJ, Priest HD, Mockler TC, et al.. Functional characterization of cinnamyl alcohol dehydrogenase and caffeic acid O-methyltransferase in Brachypodium distachyon. BMC Biotechnol. 2013;16:61

S22: Zhao Q, Tobimatsu Y, Zhou R, Pattathil S, Gallego-Giraldo L, Fu C, et al.. Loss of function of cinnamyl alcohol dehydrogenase 1 leads to unconventional lignin and a temperature-sensitive growth defect in Medicago truncatula. Proc Natl Acad Sci U S A. 2013;110:13660-13665

S23: Liu KH, Huang CY, Tsay YF. CHL1 is a dual-affinity nitrate transporter of Arabidopsis involved in multiple phases of nitrate uptake. Plant Cell. 1999;11:865-874

S24: Remans T, Nacry P, Pervent M, Girin T, Tillard P, Lepetit M, et al. A central role for the nitrate transporter NRT2.1 in the integrated morphological and physiological responses of the root system to nitrogen limitation in Arabidopsis. Plant Physiol. 2006;140:909-921

S25: Kiba T, Feria-Bourrellier AB, Lafouge F, Laxhneva L, Boutet-Mercey S, Orsel M, et al. The Arabidopsis nitrate transporter NRT2.4 plays a double role in roots and shoots of nitrogen-starved plants. Plant Cell. 2012;24:245-258

S26: Xia X, Fan X, Wei J, Feng H, Qu H, Xie D, et al. Rice nitrate transporter OsNPF2.4 functions in low-affinity acquisition and long-distance transport. J Exp Bot. 2015;66:317-331

S27: Orsel M, Chopin F, Leleu O, Smith SJ, Krapp A, Daniel-Vedele F, et al.. Characterization of a two-component high-affinity nitrate uptake system in Arabidopsis. Physiology and protein-protein interaction. Plant Physiol. 2006;142:1304-1317

S28: Tran HT, Hurley BA, Plaxton WC. Feeding hungry plants: The role of purple acid phosphatases in phosphate nutrition. Plant Sci. 2010;179:14-27

S29: Kuang R, Chan K, Yeung E, Lim BL. Molecular and biochemical characterization of AtPAP15, a purple acid phosphatase with phytase activity, in Arabidopsis. Plant Physiol. 2009;151:199-209

S30: Zhang Q, Wang C, Tian J, Li K, Shou H. Identification of rice purple acid phosphatases related to phosphate starvation signalling. Plant Biol (Stuttg). 2011;13(1):7-15

S31: Sisaphaithong T, Kondo D, Matsunaga H, Kobae Y, Hata S. Expression of plant genes for arbuscular mycorrhiza-inducible phosphate transporters and fungal vesicle formation in sorghum, barley, and wheat roots. Biosci Biotechnol Biochem. 2012;76(12):2364-2367

S32: Yang SY, Grønlund M, Jakobsen I, Grotemeyer MS, Rentsch D, Miyao A, et al.. Nonredundant regulation of rice arbuscular mycorrhizal symbiosis by two members of the phosphate transporter1 gene family. Plant Cell. 2012;10:4236-4251

S33: Paszkowski U, Kroken S, Roux C, Briggs SP. Rice phosphate transporters include an evolutionarily divergent gene specifically activated in arbuscular mycorrhizal symbiosis. Proc Natl Acad Sci U S A. 2002;99:13324-13329

S34: Loth-Pereda V, Orsini E, Courty P, Lota F, Kohler A, Diss L, et al.. Structure and expression profile of the phosphate Pht1 transporter gene family in mycorrhizal Populus trichocarpa. Plant Physiol. 2011;156:2141-2154

S35: Gu M, Chen A, Sun S, Xu G. Complex Regulation of Plant Phosphate Transporters and the Gap between Molecular Mechanisms and Practical Application: What Is Missing? Mol Plant. 2016 Mar 7;9(3):396-416.

S36: Inoue H, Kobayashi T, Nozoye T, Takahashi M, Kakei Y, Suzuki K, et al.. Rice OsYSL15 is an iron-regulated iron(III)-deoxymugineic acid transporter expressed in the roots and is essential for iron uptake in early growth of the seedlings. J Biol Chem. 2009;284:3470-3479

S37: Davletova S, Rizhsky L, Liang H, Zhong S, Oliver D, Coutu J, et al. Cytosolic ascorbate peroxidase 1 is a central component of the reactive oxygen gene network of Arabidopsis. Plant Cell. 2005;17:268-281

S38: Vietch NC. Structural determinants of plant peroxidase function. Phytochemistry Reviews, 2004; 3:3-18

S39: Cheng Q, Li N, Dong L, Zhang D, Fan S, Jiang L, Wang X, Xu P, Zhang S. Overexpression of Soybean Isoflavone Reductase (GmIFR) Enhances Resistance to Phytophthora sojae in Soybean. Front Plant Sci, 2015;6:1024

S40: Takahashi S, Yeo YS, Zhao Y, O'Maille PE, Greenhagen BT, Noel JP, et al.. Functional characterization of premnaspirodiene oxygenase, a cytochrome P450 catalyzing regio- and stereo-specific hydroxylations of diverse sesquiterpene substrates. J Biol Chem. 2007;282:31744-31754

S41: Taira T, Ohnuma T, Yamagami T, Aso Y, Ishiguro M, Ishihara M. Antifungal activity of rye (Secale cereale) seed chitinases: the different binding manner of class I and class II chitinases to the fungal cell walls. Biosci Biotechnol Biochem. 2002;66:970-977

S42: Gupta M, Qiu X, Wang L, Xie W, Zhang C, Xiong L, et al. KT/HAK/KUP potassium transporters gene family and their whole-life cycle expression profile in rice (Oryza sativa). Mol Genet Genomics. 2008;280:437-452

S43: Wang Y, Wu W. Potassium transport and signaling in higher plants. Annu. Rev. Plant Biol. 2013;64:451-476

S44: Nieves-Cordones M, Alemán F, Martínez V, Rubio F. The Arabidopsis thaliana HAK5 K+ transporter is required for plant growth and K+ acquisition from low K+ solutions under saline conditions. Mol Plant. 2010;3:326-333

S45: Sze H, Padmanaban S, Cellier F, Honys D, Cheng NH, Bock KW, et al. Expression patterns of a novel AtCHX gene family highlight potential roles in osmotic adjustment and K+ homeostasis in pollen development. Plant Physiol. 2004;136:2532-2547

S46: Ren ZH, Gao JP, Li LG, Cai XL, Huang W, Chao DY, et al. A rice quantitative trait locus for salt tolerance encodes a sodium transporter. Nat Genet. 2005;37:1141-1146

S47: Byrt CA, Platten JD, Spielmeyer W, James RA, Lagudah ES, Dennis ES, et al. HKT1;5-like cation transporters linked to Na+ exclusion loci in wheat, Nax2 and Kna1. Plant Physiol. 2007;143:1918-1928

S48: James RA, Blake C, Byrt CS, Munns R. Major genes for Na+ exclusion, Nax1 and Nax2 (wheat HKT1;4 and HKT1;5), decrease Na+ accumulation in bread wheat leaves under saline and waterlogged conditions. J Exp Bot. 2011;62:2939-2947

S49: Whitney PA, Magasanik B. The induction of arginase in Saccharomyces cerevisiae. J Biol Chem, 1973; 248(17):6197-6202

S50: Takahashi S, Kakuichi T, Fujii K, Kera Y, Yamada R. Physiological role of D-aspartate oxidase in the assimilation and detoxification of D-aspartate in the yeast Cryptococcus humicola. Yeast. 2005;22:1203-1212

S51: Paul JH, Cooksey KE. Asparagine metabolism and asparaginase activity in a euryhaline Chlamydomonas species. Can J Microbiol, 1979;25(12):1443-1451 PubMed PMID: 43771.

S52: Mimura S, Masuda T, Matsui T, Takisawa H. Central role for cdc45 in establishing an initiation complex of DNA replication in Xenopus egg extracts. Genes Cells. 2000;5:439-452

S53: Saito Y, Uraki F, Nakajima S, Asaeda A, Ono K, Kuno K, et al. Characterization of endonuclease III (nth) and endonuclease VIII (nei) mutants of Escherichia coli K-12. J Bacteriol. 1997;179:3783-3785

S54: Martin IV, MacNeill SA. ATP-dependent DNA ligases. Genome Biol. 2002;3:REVIEWS3005

S55: Lario LD, Ramirez-Parra E, Gutierrez C, Casati P, Spampinato CP. Regulation of plant MSH2 and MSH6 genes in the UV-B-induced DNA damage response. J Exp Bot. 2011;62:2925-2937

S56: Hall MC, Shcherbakova PV, Fortune JM, Borchers CH, Dial JM, Tomer KB, et al. DNA binding by yeast Mlh1 and Pms1: implications for DNA mismatch repair. Nucleic Acids Res. 2003;31:2025-2034

S57: Guzder SN, Sung P, Prakash L, Prakash S. Yeast Rad7-Rad16 complex, specific for the nucleotide excision repair of the nontranscribed DNA strand, is an ATP-dependent DNA damage sensor. J Biol Chem. 1997;272:21665-21668

S58: Shevelev IV, Hübscher U. The 3'-5' exonucleases. Nat Rev Mol Cell Biol. 2002;3:364-376

S59: Aono T, Maldonado-Mendoza I, Dewbre GR, Harrison MJ, Saito M. Expression of alkaline phosphatase genes in arbuscular mycorrhizas. New Phytol. 2004;162:525-534

S60: Della Monica IF, Saparrat MCN, Godeas AM, Scervino JM. The co-existence between DSE and AMF symbionts affects plant P pools through P mineralization and solubilization processes. Fungal Ecol. 2015;17:10-17

S61: Oddon DM, Diatloff E, Roberts SK. A CLC chloride channel plays an essential role in copper homeostasis in Aspergillus nidulans at increased extracellular copper concentrations. Biochim Biophys Acta. 2007;1768:2466-2477

S62: Philpott CC. Iron uptake in fungi: a system for every source. Biochim Biophys Acta. 2006;1763:636-645

S63: Lee JY, Yang JG, Zhitnitsky D, Lewinson O, Rees DC. Structural basis for heavy metal detoxification by an Atm1-type ABC exporter. Science. 2014;343:1133-1136

S64: Ortiz DF, Ruscitti T, McCue KF, Ow DW. Transport of metal-binding peptides by HMT1, a fission yeast ABC-type vacuolar membrane protein. J Biol Chem. 1995;270:4721-4728

S65: Kinclova-Zimmermannova O, Gaskova D, Sychrova H. The Na+,K+/H+ -antiporter Nha1 influences the plasma membrane potential of Saccharomyces cerevisiae. FEMS Yeast Res. 2006;6:792-800

S66: Kumánovics A, Poruk KE, Osborn KA, Ward DM, Kaplan J. YKE4 (YIL023C) encodes a bidirectional zinc transporter in the endoplasmic reticulum of Saccharomyces cerevisiae. J Biol Chem. 2006;281:22566-22574

S67: Aono T, Maldonado-Mendoza I, Dewbre GR, Harrison MJ, Saito M. Expression of alkaline phosphatase genes in arbuscular mycorrhizas. New Phytol. 2004;162:525-534

S68: Della Monica IF, Saparrat MCN, Godeas AM, Scervino JM. The co-existence between DSE and AMF symbionts affects plant P pools through P mineralization and solubilization processes. Fungal Ecol. 2015;17:10-17

S69: Kumánovics A, Poruk KE, Osborn KA, Ward DM, Kaplan J. YKE4 (YIL023C) encodes a bidirectional zinc transporter in the endoplasmic reticulum of Saccharomyces cerevisiae. J Biol Chem. 2006;281:22566-22574

S70: Singh S, Brocker C, Koppaka V, Ying C, Jackson B, Matsumoto A, et al.. Aldehyde dehydrogenases in cellular responses to oxidative/electrophilic stress. Free Radic Biol Med. 2013;56:89-101

S71: Asiimwe T, Krause K, Schlunk I, Kothe E. Modulation of ethanol stress tolerance by aldehyde dehydrogenase in the mycorrhizal fungus Tricholoma vaccinum. Mycorrhiza. 2012;22:471-484

S72: Cai Z, Peng G, Cao Y, Liu Y, Jin K, Xia Y. Trehalose-6-phosphate synthase 1 from Metarhizium anisopliae: clone, expression and properties of the recombinant. J Biosci Bioeng. 2009;107:499-505

S73: Elbein A, Pan Y, Pastuszak I, Carroll D. New insights on trehalose: a multifunctional molecule. Glycobiology. 2003;13:17R-27R

S74: Jain NK, Roy I. Trehalose and protein stability. Curr Protoc Protein Sci. 2010; doi:10.1093/glycob/cwg047.

S75: Wang Y, Liu T, Patel S, Jiang L, Xue C. The casein kinase I protein Cck1 regulates multiple signaling pathways and is essential for cell integrity and fungal virulence in Cryptococcus neoformans. Eukaryot Cell. 2011;10:1455-1464

S76: Paris S, Wysong D, Debeaupuis J, Shibuya K, Philippe B, Diamond RD, et al. Catalases of Aspergillus fumigatus. Infect Immun. 2003;71:3551-3562

S77: Yao SH, Guo Y, Wang YZ, Zhang D, Xu L, Tang WH. A cytoplasmic Cu-Zn superoxide dismutase SOD1 contributes to hyphal growth and virulence of Fusarium graminearum. Fungal Genet Biol. 2016;91:32-42

S78: Zhang W, Hanisch S, Kwaaitaal M, Pedersen C, Thordal-Christensen H. A component of the Sec61 ER protein transporting pore is required for plant susceptibility to powdery mildew. Front Plant Sci. 2013;16:127

S79: Yao SH, Guo Y, Wang YZ, Zhang D, Xu L, Tang WH. A cytoplasmic Cu-Zn superoxide dismutase SOD1 contributes to hyphal growth and virulence of Fusarium graminearum. Fungal Genet Biol. 2016;91:32-42

S80: Abbà S, Khouja HR, Martino E, Archer DB, Perotto S. SOD1-targeted gene disruption in the ericoid mycorrhizal fungus Oidiodendron maius reduces conidiation and the capacity for mycorrhization. Mol Plant Microbe Interact. 2009;22:1412-1421
