# Supplementary material for: Microbiome and ecotypic adaption of Holcus lanatus (L.) to extremes of its soil pH range, investigated through transcriptome sequencing
Source: Microbiome. 2018 Mar 20;6:48. doi: 10.1186/s40168-018-0434-3 (PMC5859661; doi:10.1186/s40168-018-0434-3)
Supplement: Supplementary file 10 — Additional qPCR information. Targets and primers used for qPCR and comparison of results obtained with RNA-Seq and qPCR. (DOCX 22 kb) [file 40168_2018_434_MOESM10_ESM.docx]

**Additional file 10:** Additional qPCR information.

Details of target genes and primers used for qPCR.

| \| **Assembly and Trinity ID** \| **Plant-refseq annotation** \| **Primer sequence (5' - 3')** \| **Amplicon size (bp)** \| \| --- \| --- \| --- \| --- \| \| A-TR35403\|c1_g1_i1 \| Two pore potassium channel a \| F: TTCGATCACTATCCAGATCAGC \| 127 \| \| R: GCACTATTCTTCATGTACCTTGC \| \| B-TR61836\|c0_g1_i1 \| Adenylosuccinate synthetase \| F: ACTTCTTCAAGCCCTTCTGC \| 134 \| \| R: AGCAGTTCTGTTGGGAAAGG \| \| G-TR51302\|c0_g1_i1 \| 2-component response regulator PRR95 \| F: AGGTTGCTGGACAGATAATGG \| 107 \| \| R: AGTTGTTGGTTGGGAAGTAGG \| \| E-TR42208\|c1_g1_i1 \| Hydroxycinnamoyl-Coenzyme A shikimate/quinate hydroxycinnamoyltransferase \| F: GACACATACACATTCACCAAATCC \| 102 \| \| R: GGAGTGTGATCTTCACTAGATCG \| \| housekeeping gene for normalisation [74] \| AK059783 18s ribosomal RNA \| F: CTACGTCCCTGCCCTTTGTACA \| 65 \| \| R: ACACTTCACCGGACCATTCAA \| |
| --- | --- | --- | --- | --- | --- | --- | --- | --- | --- | --- | --- | --- | --- | --- | --- | --- | --- | --- | --- | --- | --- | --- | --- | --- | --- | --- | --- | --- | --- |

Comparison of results obtained for RNA-Seq and qPCR in RNA extracted from shoot of *Holcus lanatus* plants.

| \|  \|  \| **RNA-Seq** \| \| \| **qPCR** \| \| \| \| --- \| --- \| --- \| --- \| --- \| --- \| --- \| --- \| \| **Gene** \| **Comparison** \| **log_2_ FC** \| ***p*-value** \| **FDR** \| **log_2_ FC** \| **T-value** \| **Tukey *p*-value** \| \| A-TR35403\|c1_g1_i1 \| Al v Ll \| 0.51 \| N.S \| N.S \| 0.56 \| -1.66 \| N.S \| \| B-TR61836\|c0_g1_i1 \| Al v Ll \| -1.31 \| <0.001 \| <0.001 \| -1.66 \| 2.95 \| <0.05 \| \| G-TR51302\|c0_g1_i1 \| Al v Ll \| -1.44 \| <0.001 \| <0.01 \| -1.6 \| 4.94 \| <0.01 \| \| E-TR42208\|c1_g1_i1 \| Al v Ll \| 0.68 \| N.S \| N.S \| 0.72 \| -2.02 \| N.S \| \| A-TR35403\|c1_g1_i1 \| Aa v La \| 1.97 \| <0.001 \| <0.001 \| 2.13 \| -6.06 \| <0.001 \| \| B-TR61836\|c0_g1_i1 \| Aa v La \| -1.82 \| <0.001 \| <0.001 \| -1.74 \| 3.93 \| <0.01 \| \| G-TR51302\|c0_g1_i1 \| Aa v La \| -2.08 \| <0.001 \| <0.001 \| -2.06 \| 6.37 \| <0.001 \| \| E-TR42208\|c1_g1_i1 \| Aa v La \| 2.32 \| <0.001 \| <0.001 \| 2.2 \| -6.22 \| <0.001 \| \| A-TR35403\|c1_g1_i1 \| Aa v Al \| 0.80 \| <0.05 \| N.S \| 0.91 \| -2.29 \| N.S \| \| B-TR61836\|c0_g1_i1 \| Aa v Al \| -0.30 \| N.S \| N.S \| -0.13 \| 0.36 \| N.S \| \| G-TR51302\|c0_g1_i1 \| Aa v Al \| -0.72 \| <0.05 \| N.S \| -0.67 \| 1.64 \| N.S \| \| E-TR42208\|c1_g1_i1 \| Aa v Al \| 0.58 \| N.S \| N.S \| 0.75 \| -1.93 \| N.S \| \| A-TR35403\|c1_g1_i1 \| La v Ll \| -0.66 \| N.S \| N.S \| -0.65 \| 1.9 \| N.S \| \| B-TR61836\|c0_g1_i1 \| La v Ll \| 0.21 \| N.S \| N.S \| -0.06 \| -0.42 \| N.S \| \| G-TR51302\|c0_g1_i1 \| La v Ll \| -0.08 \| N.S \| N.S \| -0.2 \| 0.48 \| N.S \| \| E-TR42208\|c1_g1_i1 \| La v Ll \| -1.06 \| <0.01 \| N.S \| -0.73 \| 2.02 \| N.S \| |
| --- | --- | --- | --- | --- | --- | --- | --- | --- | --- | --- | --- | --- | --- | --- | --- | --- | --- | --- | --- | --- | --- | --- | --- | --- | --- | --- | --- | --- | --- | --- | --- | --- | --- | --- | --- | --- | --- | --- | --- | --- | --- | --- | --- | --- | --- | --- | --- | --- | --- | --- | --- | --- | --- | --- | --- | --- | --- | --- | --- | --- | --- | --- | --- | --- | --- | --- | --- | --- | --- | --- | --- | --- | --- | --- | --- | --- | --- | --- | --- | --- | --- | --- | --- | --- | --- | --- | --- | --- | --- | --- | --- | --- | --- | --- | --- | --- | --- | --- | --- | --- | --- | --- | --- | --- | --- | --- | --- | --- | --- | --- | --- | --- | --- | --- | --- | --- | --- | --- | --- | --- | --- | --- | --- | --- | --- | --- | --- | --- | --- | --- | --- | --- | --- | --- | --- | --- | --- | --- | --- | --- | --- | --- | --- | --- |
