# Supplementary material for: Microbiome and ecotypic adaption of Holcus lanatus (L.) to extremes of its soil pH range, investigated through transcriptome sequencing
Source: Microbiome. 2018 Mar 20;6:48. doi: 10.1186/s40168-018-0434-3 (PMC5859661; doi:10.1186/s40168-018-0434-3)
Supplement: Supplementary file 14 — Significantly expressed and KOG annotated Glomeromycotina transcripts in root and shoot in each soil. The number of significantly expressed and KOG annotated Glomeromycotina transcripts (All) is defined as the number of transcripts that obtained ≥5 aligned reads in at least 3 samples from each of the following treatments: root acid bog soil (RA), root lime soil (RL), shoot acid bog soil (SA) and shoot lime soil (SL). Significant ecotype effects (absolute log2FC ≥ 1 or ≤ − 1, FDR < 0.05) as identified by DESeq2 analysis are reported in subsequent columns for each treatment group, under the heading plant effect. (DOCX 27 kb) [file 40168_2018_434_MOESM14_ESM.docx]

**Additional file 14:** Significantly expressed and KOG annotated Basidiomycete transcripts in roots and shoots in each soil. The number of significantly expressed and KOG annotated Basidiomycete transcripts (All) is defined as the number of transcripts that obtained ≥ 5 aligned reads in at least 3 samples from each of the following treatments: root acid bog soil (RA), root lime soil (RL), shoot acid bog soil (SA) and shoot lime soil (SL). Significant ecotype effects (absolute log_2_FC ≥ 1 or ≤ -1, FDR < 0.05) as identified by DESeq2 analysis are reported in subsequent columns for each treatment group, under the heading plant effect.

| Basidiomycetes | **SA** expressed at ≥ 5 reads in 3 out of 10 samples | | | **SL** expressed at ≥ 5 reads in 3 out of 10 samples | | | **RA** expressed at ≥ 5 reads in 3 out of 10 samples | | | **RL** expressed at ≥ 5 reads in 3 out of 10 samples | | |
| --- | --- | --- | --- | --- | --- | --- | --- | --- | --- | --- | --- | --- |
|  | All | Acid plant | Lime plant | All | Acid plant | Lime plant | All | Acid plant | Lime plant | All | Acid plant | Lime plant |
| CELLULAR PROCESSES AND SIGNALING | 7 | 0 | 0 | 7 | 0 | 0 | 100 | 0 | 17 | 55 | 1 | 4 |
| Cell motility | 0 | 0 | 0 | 0 | 0 | 0 | 0 | 0 | 0 | 0 | 0 | 0 |
| Cell wall/membrane/envelope biogenesis | 0 | 0 | 0 | 0 | 0 | 0 | 6 | 0 | 1 | 0 | 0 | 0 |
| Cytoskeleton | 5 | 0 | 0 | 5 | 0 | 0 | 26 | 0 | 7 | 19 | 0 | 2 |
| Defense mechanisms | 0 | 0 | 0 | 0 | 0 | 0 | 0 | 0 | 0 | 0 | 0 | 0 |
| Extracellular structures | 0 | 0 | 0 | 0 | 0 | 0 | 0 | 0 | 0 | 0 | 0 | 0 |
| Intracellular trafficking, secretion, and vesicular transport | 0 | 0 | 0 | 0 | 0 | 0 | 7 | 0 | 0 | 4 | 0 | 1 |
| Nuclear structure | 0 | 0 | 0 | 0 | 0 | 0 | 0 | 0 | 0 | 0 | 0 | 0 |
| Posttranslational modification, protein turnover, chaperones | 2 | 0 | 0 | 2 | 0 | 0 | 47 | 0 | 6 | 22 | 1 | 1 |
| Signal transduction mechanisms | 0 | 0 | 0 | 0 | 0 | 0 | 14 | 0 | 3 | 10 | 0 | 0 |
|  |  |  |  |  |  |  |  |  |  |  |  |  |
| INFORMATION STORAGE AND PROCESSING | 4 | 0 | 0 | 3 | 0 | 0 | 85 | 2 | 25 | 56 | 0 | 2 |
| Chromatin structure and dynamics | 0 | 0 | 0 | 0 | 0 | 0 | 1 | 0 | 0 | 2 | 0 | 0 |
| Replication, recombination and repair | 0 | 0 | 0 | 0 | 0 | 0 | 1 | 0 | 0 |  | 0 | 0 |
| RNA processing and modification | 0 | 0 | 0 | 0 | 0 | 0 | 6 | 0 | 0 | 3 | 0 | 0 |
| Transcription | 0 | 0 | 0 | 0 | 0 | 0 | 4 | 0 | 1 |  | 0 | 0 |
| Translation, ribosomal structure and biogenesis | 4 | 0 | 0 | 3 | 0 | 0 | 73 | 2 | 24 | 51 | 0 | 2 |
|  |  |  |  |  |  |  |  |  |  |  |  |  |
| METABOLISM | 2 | 0 | 0 | 2 | 0 | 0 | 92 | 1 | 19 | 21 | 0 | 0 |
| Amino acid transport and metabolism | 0 | 0 | 0 | 0 | 0 | 0 | 18 | 0 | 2 | 0 | 0 | 0 |
| Carbohydrate transport and metabolism | 0 | 0 | 0 | 0 | 0 | 0 | 10 | 0 | 4 | 4 | 0 | 0 |
| Cell cycle control, cell division, chromosome partitioning | 0 | 0 | 0 | 0 | 0 | 0 | 3 | 0 | 0 | 0 | 0 | 0 |
| Coenzyme transport and metabolism | 1 | 0 | 0 | 1 | 0 | 0 | 4 | 0 | 1 | 2 | 0 | 0 |
| Energy production and conversion | 1 | 0 | 0 | 1 | 0 | 0 | 39 | 0 | 11 | 14 | 0 | 0 |
| Inorganic ion transport and metabolism | 0 | 0 | 0 | 0 | 0 | 0 | 8 | 1 | 0 | 0 | 0 | 0 |
| Lipid transport and metabolism | 0 | 0 | 0 | 0 | 0 | 0 | 7 | 0 | 0 | 1 | 0 | 0 |
| Nucleotide transport and metabolism | 0 | 0 | 0 | 0 | 0 | 0 | 3 | 0 | 1 | 0 | 0 | 0 |
| Secondary metabolites biosynthesis, transport and catabolism | 0 | 0 | 0 | 0 | 0 | 0 | 0 | 0 | 0 | 0 | 0 | 0 |
|  |  |  |  |  |  |  |  |  |  |  |  |  |
| POORLY CHARACTERIZED | 0 | 0 | 0 | 0 | 0 | 0 | 14 | 0 | 0 | 0 | 0 | 0 |
| Function unknown | 0 | 0 | 0 | 0 | 0 | 0 | 2 | 0 | 0 | 0 | 0 | 0 |
| General function prediction only | 0 | 0 | 0 | 0 | 0 | 0 | 12 | 0 | 0 | 0 | 0 | 0 |
|  |  |  |  |  |  |  |  |  |  |  |  |  |
| Total KOG annotated | 13 | 0 | 0 | 12 | 0 | 0 | 291 | 3 | 61 | 132 | 1 | 6 |
