# Supplementary material for: Microbiome and ecotypic adaption of Holcus lanatus (L.) to extremes of its soil pH range, investigated through transcriptome sequencing
Source: Microbiome. 2018 Mar 20;6:48. doi: 10.1186/s40168-018-0434-3 (PMC5859661; doi:10.1186/s40168-018-0434-3)
Supplement: Supplementary file 16 — Colonization percentages of AM hyphae, non-AM hyphae, arbuscules and vesicles, in acid bog and limestone quarry ecotypes of H. lanatus, grown in a reciprocal soil transplantation design. (A = acid bog soil, L = limestone quarry soil), plant ecotype (a = acid bog plant ecotype, l = limestone quarry plant ecotype). (DOCX 18 kb) [file 40168_2018_434_MOESM16_ESM.docx]

| **Structure** | **Mean % infection** | | | | **Standard error** | | | |
| --- | --- | --- | --- | --- | --- | --- | --- | --- |
|  | **Aa** | **Al** | **La** | **Ll** | **Aa** | **Al** | **La** | **Ll** |
| AM hyphae | 13.5 | 13.7 | 49 | 44.5 | 2.5 | 1.7 | 3.7 | 6.8 |
| Non-AM hyphae | 32.5 | 53.8 | 12.2 | 10.7 | 2.3 | 5.9 | 1.7 | 2.8 |
| Arbuscules | 4.3 | 5.2 | 17 | 13.3 | 1.3 | 0.8 | 1.9 | 2.4 |
| Vesicles | 0 | 0 | 2.2 | 1.7 | 0 | 0 | 0.3 | 0.8 |
| **Structure** | **Soil effect** | | **Ecotype effect** | | **Interaction** | |  |  |
|  | **F-value** | ***p*-value** | **F-value** | ***p*-value** | **F-value** | ***p*-value** |  |  |
| AM hyphae | 65.51 | **< 0.001** | 0.53 | 0.473 | --- | --- |  |  |
| Non-AM hyphae | 72.66 | **< 0.001** | 0.61 | 0.444 | 6.06 | **< 0.05** |  |  |
| Arbuscules | 37.05 | **< 0.001** | 0.05 | 0.82 | --- | --- |  |  |
| Vesicles | 43.08 | **< 0.001** | 1.72 | 0.203 | --- | --- |  |  |

**Additional file 16:** Colonisation percentages of AM hyphae, non-AM hyphae, arbuscules and vesicles, in acid bog and limestone quarry ecotypes of *H. lanatus*, grown in a reciprocal soil transplantation design. (*A* = acid bog soil, *L* = limestone quarry soil), plant ecotype (*a* = acid bog plant ecotype, *l* = limestone quarry plant ecotype).
