# Supplementary material for: Microbiome and ecotypic adaption of Holcus lanatus (L.) to extremes of its soil pH range, investigated through transcriptome sequencing
Source: Microbiome. 2018 Mar 20;6:48. doi: 10.1186/s40168-018-0434-3 (PMC5859661; doi:10.1186/s40168-018-0434-3)
Supplement: Supplementary file 17 — AM and non-AM fungal structures of stained H. lanatus roots of (a, b) acid bog ecotype on acid bog soil; (c, d) limestone quarry ecotype on acid bog soil; (e, f) acid bog ecotype on limestone quarry soil; (g, h) limestone quarry ecotype on limestone quarry soil. (DOCX 93871 kb) [file 40168_2018_434_MOESM17_ESM.docx]

**Additional file 17:** AM and non-AM fungal structures of stained *H. lanatus* roots of (a, b) acid bog ecotype on acid bog soil; (c, d) limestone quarry ecotype on acid bog soil; (e, f) acid bog ecotype on limestone quarry soil; (g, h) limestone quarry ecotype on limestone quarry soil.
